# The adaptive landscape of wildtype and glycosylation-deficient populations of the industrial yeast *Pichia pastoris*

**DOI:** 10.1186/s12864-017-3952-7

**Published:** 2017-08-10

**Authors:** Josef W. Moser, Iain B. H. Wilson, Martin Dragosits

**Affiliations:** 1 Department of Chemistry, University of Natural Resources and Life Sciences, Muthgasse 18, 1190 Vienna, Austria; 2 Austrian Centre of Industrial Biotechnology (ACIB), Muthgasse 11, 1190 Vienna, Austria

**Keywords:** *Pichia Pastoris*, Experimental evolution, Glucose, Salt stress, *OCH1*

## Abstract

**Background:**

The effects of long-term environmental adaptation and the implications of major cellular malfunctions are still poorly understood for non-model but biotechnologically relevant species. In this study we performed a large-scale laboratory evolution experiment with 48 populations of the yeast *Pichia pastoris* in order to establish a general adaptive landscape upon long-term selection in several glucose-based growth environments. As a model for a cellular malfunction the implications of *OCH1* mannosyltransferase knockout-mediated glycosylation-deficiency were analyzed.

**Results:**

In-depth growth profiling of evolved populations revealed several instances of genotype-dependent growth trade-off/cross-benefit correlations in non-evolutionary growth conditions. On the genome level a high degree of mutational convergence was observed among independent populations. Environment-dependent mutational hotspots were related to osmotic stress-, Rim - and cAMP signaling pathways. In agreement with the observed growth phenotypes, our data also suggest diverging compensatory mutations in glycosylation-deficient populations. High osmolarity glycerol (HOG) pathway loss-of-functions mutations, including genes such as *SSK2* and *SSK4,* represented a major adaptive strategy during environmental adaptation. However, genotype-specific HOG-related mutations were predominantly observed in opposing environmental conditions. Surprisingly, such mutations emerged during salt stress adaptation in *OCH1* knockout populations and led to growth trade-offs in non-adaptive conditions that were distinct from wildtype HOG-mutants. Further environment-dependent mutations were identified for a hitherto uncharacterized species-specific Gal4-like transcriptional regulator involved in environmental sensing.

**Conclusion:**

We show that metabolic constraints such as glycosylation-deficiency can contribute to evolution on the molecular level, even in non-diverging growth environments. Our dataset suggests universal adaptive mechanisms involving cellular stress response and cAMP/PKA signaling but also the existence of highly species-specific strategies involving unique transcriptional regulators, improving our biological understanding of distinct *Ascomycetes* species.

**Electronic supplementary material:**

The online version of this article (doi:10.1186/s12864-017-3952-7) contains supplementary material, which is available to authorized users.

## Background

Microbial cells have to maintain a viable state in a wide variety of environmental conditions. They are exposed to many fluctuating and stressful conditions such as nutrient limitation and changes of temperature, pH or osmotic strength. Thus, the general strategies and gene regulatory mechanisms towards environmental change are well-established for both, pro- and eukaryotic organisms. Previous research showed that short and long-term adaptation to a specific environmental trait does not necessarily lead to growth trade-offs in other adversarial environmental conditions but that certain abiotic stress combinations can lead to cross-protection [1–4].

In this context, biological systems are robust towards perturbation on multiple levels such as environmental change and genetic mutations [5]. Furthermore, the gene regulatory networks underlying the physiological response possess a high degree of topological robustness. Cellular networks are essentially scale-free networks with most nodes having few interaction partners; only a minor fraction of proteins has multiple interaction partners or regulatory connections and serve as so-called network hubs [6]. The removal of hubs from any regulatory network is more likely to lead to lethal phenotypes than the removal of sparsely connected nodes [7, 8]. Such hubs are well conserved across different species [6]. Nevertheless, biological networks have been previously shown to tolerate the addition of novel nodes and large-scale rewiring, thereby establishing novel interactions and driving the evolution of regulatory networks [9, 10]. Considering the implications of long-term divergent environmental conditions in the incipient processes of speciation [11], these mechanisms also constitute the basis for the differences in regulatory patterns among different species that are observed today [12].

**Fig. 1:**
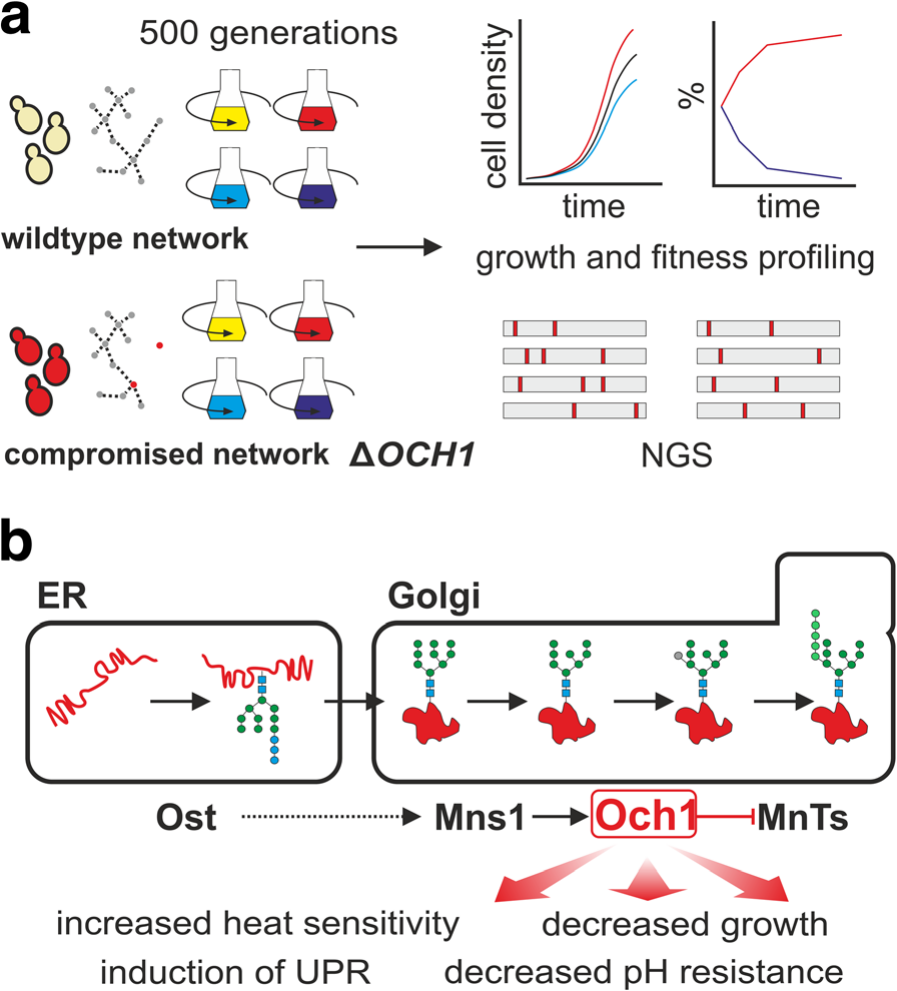
Experimental setup. **a** Experimental setup to analyze consequences of environmental adaptation in *P. pastoris* wildtype and mutant (∆*OCH1*) populations. Wildtype populations and glycosylation-deficient populations were propagated for 500 generations by serial transfers and subsequently analyzed in terms of growth rates and competitive fitness on the population and single clone level, followed by next generation sequencing (NGS) of selected clones. **b** Yeast protein N-glycosylation pathway. The Och1 mannosyltransferase mediates the transfer of a mannose moiety to the precursor N-glycan and thereby initiates outer chain elongation and hypermannosylation in yeasts. The prevention of this step by *OCH1* deletion results in several growth defects. Ost – Oligosaccharyltransferases, Mns1 – Mannosidase 1, Och1 – outer chain elongation factor 1, MnTs – Mannosyltransferases

Whereas previous studies analyzed the adaptive trajectories of model organisms such as *E. coli* and *S. cerevisiae* on an evolutionary scale [4, 13, 14] and showed that microbial cells can develop compensatory mutations during experimental evolution when important nodes are removed [15–18], little is known for non-model microbial species. Towards this end, we performed the first large-scale laboratory evolution study on the methylotrophic yeast *Pichia pastoris* (*Komagataella phaffii*) [19]*,* an important recombinant protein production host organism [20], in order to analyze evolutionary trade-off and cross-benefit effects in nutrient-rich and nutrient-poor growth media with glucose as carbon source (Fig. 1a).

Furthermore, we analyzed the impact of non-lethal node removal on the evolutionary trajectories of evolving *P. pastoris* populations, using the biotechnologically important *OCH1* gene knockout as model system (Fig. 1b): The modification of the cellular protein-N-glycosylation machinery to promote the formation of truncated or humanized N-glycans is of great interest in fungal biotechnology [21, 22]. A prerequisite for such glycan-remodeling is the prevention of mannose-chain elongation during yeast N-glycan maturation. This is achieved knocking out the *OCH1* mannosyltransferase. The removal of this node leads to the formation of abnormal, truncated, N-glycan structures (Fig. 1b). Protein glycosylation is important for protein maturation and stability [23] and subsequently for efficient growth. Therefore *OCH1* node removal, although leading to a viable phenotype, has already been previously reported to lead to abnormal growth and stress resistance in yeasts [21, 24]. As a consequence, attempts have already been made to improve growth of glyco-engineered yeasts [25].

In the current study, after 500 generations of laboratory evolution of 48 *P. pastoris* wildtype and *∆OCH1* populations, growth rates and competitive fitness of ancestral and evolved populations were analyzed to establish a trade-off cross-benefit correlation map. Subsequently, genome sequencing was performed in order to identify environment- and genotype-specific mutations underlying the improved phenotypes.

## Methods

### Yeast strains

*P. pastoris* X-33, BG10 and BG10 *∆OCH1* strains were used for all experiments. Strains BG10 and BG10 *∆OCH1* (BSY 10 M81) were obtained from bisy e.U. (Austria); to allow direct competition assays of different populations, strains were tagged with fluorescent gene markers. Glycerol stocks from the strains X-33, BG10 and BG10 ∆*OCH1* were streaked on YPD (Yeast extract, peptone 2% glucose, pH 7.4) agar plates and grown at 28 °C for 48 h. A single colony from each strain was used to prepare electro-competent cells as described previously [26] and transformed with SacI (New England Biolabs) linearized plasmids containing either an green fluorescent protein (eGFP) or red fluorescent protein (DsRED) expression cassette under the control of a constitutive promoter, P_*PGK1*_ (Additional file 1: Fig. S1). From each strain – fluorescent marker combination, a single random clone with verified fluorescence protein expression was chosen as founding clone for the evolving populations.

### Experimental evolution

Adaptation was performed with four founding populations of each strain (X-33, BG10 and BG10 *∆OCH1,* respectively) in 4 different growth environments, resulting in 48 populations. Two GFP-tagged and two DsRED-tagged populations were used for each condition and strain. Populations were cultivated in a volume of 2 mL in 24-deep well plates (polypropylene Uniplates, cylindrical, round bottom wells with 10 mL total volume, Whatman) covered with AeraSeal gas permeable sealing films (Excel Scientific) in either YPD (Yeast extract, peptone 2% glucose, pH 7.4), YPDN (YPD, 500 mM NaCl, pH 7.4), BMD (buffered minimal medium: 100 mM potassium phosphate, pH 6.0, 1.34% YNB, 4.5 × 10^−5^% biotin, 2% glucose) or BMDN (BMD, 250 mM NaCl) growth media at 28 °C, 200 rpm on an orbital shaker (orbit diameter 1.9 cm). The starting optical density (OD_600_) for the initial inoculation was OD_600_ = 0.1. For each passage, growth medium-filled plates were incubated at room temperature over night before use to ensure transfer into sterile medium. Populations were inoculated in a checkerboard pattern (i.e. alternating eGFP and DsRED-tagged strains) and culture fluorescence was regularly monitored to rule out lateral cross-contamination during daily transfers. Passages were performed at 1:100 dilutions every 24 h until 500 generations were reached. OD_600_ were monitored on a daily basis in order to calculate the cumulative number of cell divisions (CCD) and cumulative generations. Cultures were checked for contamination by plating samples on YPD agar plates (incubation at 30 °C for 48 h) and microscopy every 50 generations. Cryo-stocks of all populations were prepared in 50 generations intervals. All OD_600_ and fluorescent (eGfp and DsRED fluorescence) measurements were performed in 96-well plates (flat-bottom transparent for OD_600_ and flat-bottom black for fluorescence detection) on an Infinite M200 multimode plate reader (Tecan).

### Growth tests and competition assays

For growth profiling deep well plates (polypropylene Uniplates with cylindrical round bottom wells of 10 mL total volume, Whatman) covered with AeraSeal gas permeable sealing film (Excel Scientific) were applied, using 2 mL culture volume in the respective growth medium at 28 °C, 200 rpm. Starter cultures were grown for 16 to 18 h. On the next day, 2 mL cultures were inoculated at a starting OD_600_ of 0.05–0.1 and growth was monitored for 8–10 h with 1 to 2 h intervals. The maximum growth rate [μ_max_] was calculated based for the exponential growth phase (typically between time = 2 and 8 h) and final optical density (OD_600_) was determined after 24 h. To estimate the growth performance during the early growth phase after inoculation (lag-phase), the growth rate between time = 0 and 2 h was calculated. Growth media were used as described above. For growth analysis using alternative carbon sources, 2% glucose was replaced with 1% glycerol or 1% methanol.

For direct competition assays starter cultures were grown as described above and competing eGFP- and DsRED-populations were inoculated at a starting OD_600_ of 0.1 in approximately 1:1 ratios. Samples were taken at 0 h and after 24 h of growth on an orbital shaker at 200 rpm, 28 °C as described above and frozen at −80 °C until analysis. All OD_600_ measurements for 0 and 24 h samples were performed in transparent flat-bottom 96-well plates on an Infinite M200 multimode plate reader (Tecan). Frozen samples were analyzed by flow cytometry on a Gallios flow cytometer (Beckman Coulter). For each sample, 3000–10,000 cell counts were acquired and competing strains were separated based on their FL1- and FL3 fluorescent signals. Fitness was essentially calculated as described previously by Lenski and co-workers: Based on cell counts and OD_600_ data the cell density for each strain was calculated at time 0 and 24 h (c_t0_ and c_t24_). The realized Malthusian parameter (*m*) for each competing strain was calculated, with *m* = (ln c_t24_ / c_t0_). Relative fitness (ω) was defined as the ratio of *m* for each strain: ω = *m*_strain A_ / *m*_strain B_. The ancestral strains were competed in all growth media and the fitness values of evolved populations and single clones were corrected for initial fitness differences to reflect the actual increase or decrease of fitness.

### Genome sequencing

Three random colonies were picked from each population and tested for growth rate in duplicate as described above. The clone with the highest growth rate compared to the ancestor was used for genomic sequencing. 4 mL cultures of the selected clones were grown in YPD medium at 28 °C, 200 rpm for 18 h. Cells were harvested and genomic DNA was isolated using a Masterpure™ Yeast DNA purification kit (Epicentre). Illumina MiSeq paired-end sequencing with 300 bp read length was performed by the Eurofins genomics NGS laboratory (Ebersberg, Germany) using chemistry v3. The ancestral X-33 strain as sequenced in a previous study [27] was used as reference strain.

### Sequence data analysis

The raw reads were pre-processed with Cutadapt [28] by removing Illumina Universal Adapter regions, setting the minimum Phred score to 20 and minimum read length to 50 bp. The genomic sequence of the ancestral strain, used for variant detection, was obtained through de-novo assembly and grouping of the assembled scaffolds into chromosomes by mapping them on the CBS 7435 reference strain [29]. De-novo assembly was accomplished with Meraculous [30]. Input data consisted of 4,048,141 quality trimmed reads with an average read length of 260.48 bp and a mean quality Phred score of 34.0. The final assembly consisted of 48 scaffolds with a total length of 9,310,711 bp. Four scaffolds exceeded the length of 910,678 bp, which represented the N50 value. 88.47% of the input reads were used in the assembly. The mean coverage of the de-novo assembled scaffolds amounted to 97.6.

Using CONTIGuator [31] the assembled scaffolds were ordered and assigned to chromosomes of the reference genome, which allowed a rough assessment of the assembly regarding completeness. 44 scaffolds were related to one of the four chromosomes. Three scaffolds with 2506 bp, 1439 bp, and 1035 bp length respectively were not allocated. One scaffold with length 5386 bp length was identified as contamination and removed from the analysis. Gene prediction was performed with the gene finder Augustus [32]. Predicted regions were annotated by local alignment against the CBS 7435 reference strain using blast.

Single nucleotide polymorphism (SNP) and insertion/deletion (indel) detection was performed by utilizing two different strategies. Initially, a k-mer based analysis was performed with kSNP3 [33]. The optimal k-mer size was estimated using Kchooser from kSNP3. As a second approach SNPs where detected with the Genome Analysis Toolkit [34] by aligning reads against the reference assembly using bwa-mem [35] in paired-end mode. The aligned reads were further deduplicated with picard-tools and a local realignment around Indels was performed with GATK-tools. SNPs called with GATKs HaplotypeCaller tool were filtered applying default parameters. Only SNPs/indels detected with both methods were used as final results. SNP/indel locations called from reads of the assembly strain were considered as false-positive and removed. The remaining hits were manually reviewed with a genome browser.

With cn.mops [36] and Magnolya [37] two different approaches to detect potential copy number variations (CNVs) were applied. cn.mops was chosen due to its applicability for haploid organisms. Magnolya, which uses co-assemblies of individual genomes, was used as an alternative method, since it is based on co-assemblies and its results are not affected by a reference sequence. No CNVs were identified by either of the two approaches.

All Illumina MiSeq sequencing data are available from the NCBI Sequence Read Archive, Bioproject accession number PRJNA360593.

### Real-time PCR

For real-time PCR, selected clones were grown o/n in YPD medium over night as described above. Main 10 mL YPD cultures in 125 mL shake flasks were inoculated at an OD_600_ = 0.1 and grown to exponential growth phase at 28 °C, 200 rpm for 6 h. Cells were harvested by centrifugation (3500 g, 4 °C, 5 min) and stored at −80 °C until further use. Samples were combined with 500 μL acid washed glass beads (710 – 1180 μm, Sigma Adlrich), resuspended in 1 mL Tri Reagent (Sigma Aldrich) and broken in a Fastprep-24 homogenizer (MP Biomedicals) by applying two 30s 6ms^−1^ cycles. Further Tri Reagent – chloroform based RNA purification was performed according to the manufacturer’s guidelines. cDNA synthesis was performed using Superscript III reverse transcriptase (Thermo Scientific). Real-time PCR was performed using a GoTaq qPCR mastermix (Promega) on a Rotorgene Q instrument (Qiagen) using DNA oligonucleotides listed in Additional file 1: Table S1. Each sample was measured in technical quadruplicates.

### Molecular cloning and *P. pastoris* overexpression strains

*E. coli* JM109 was used for all cloning steps. PCR steps were performed using Q5 polymerase. Initial plasmid construction for reporter gene expression was performed by conventional restriction digest – ligation protocols. Promoter and reporter gene sequences were amplified with primers listed in Additional file 1: Table S1. For the construction of *ACS1*, *TUP1, FLC2* and open reading frame PAS_chr3_0669 overexpression strains, genes were PCR amplified with gene-specific primers using Q5 polymerase and ligated into EcoRI-NotI linearized pGAPzB vector using a HIFI DNA assembly mastermix (New England Biolabs). Constructs were verified by Sanger sequencing and after linearization with MfeI used for transformation of competent *P. pastoris* X-33, BG10 and BG10 *∆OCH1* cells. Q5 polymerase, T4 DNA ligase and Hifi DNA assembly mastermix were obtained from New England Biolabs. Primers used for amplification are listed in Additional file 1: Table S1. Growth tests for overexpressing and corresponding empty vector control strains were performed as described above.

## Results

### Experimental design and growth characteristics of the ancestral strains

We present the first large-scale experimental evolution study with *P. pastoris* as a model organism. Methanol metabolism and methanol-based bioprocesses represent a hallmark of *P. pastoris* [38, 39], and recently in a smaller setup, the adaptive paths during methanol adaption were analyzed [27]. In the current study, we focused on glucose as carbon source, since glucose-based recombinant protein bioprocesses are also widely applied for this yeast [40] and glucose-adaptation facilitates comparison with results from non-methylotrophic species.

Three different *P. pastoris* strains (wildtype X-33 and BG10 and a BG10 ∆*OCH1* strain) were the starting point for the evolution experiment in different environmental conditions. Two distinct wildtype strains were included in order to evaluate the consistency of the evolutionary trajectories on a phenotypic and genomic level in different strain backgrounds. Growth media commonly used for *P. pastoris* propagation and recombinant protein production were applied: YPD medium (glucose-based rich medium) is widely used for various yeast species and supports fast growth rates and usually high recombinant protein titers in small-scale *P. pastoris* cultures. BMD (glucose-based buffered minimal medium [41] is a growth medium that is widely used for recombinant protein production as its minimal composition facilitates downstream processing and also resembles growth media that are used for larger scale biotechnological processes. Both growth media were also used with increased salt concentrations because of the implications of high salt concentrations (e.g. fed batch processes) during recombinant protein production [42] and previous system level analysis of salt stress in *P. pastoris* [43]. 500 mM NaCl were applied in YPD conditions (YPDN) and 250 mM NaCl were used for BMD conditions (BMDN) as initial growth tests with the ancestral X-33 strain suggested similar growth rate reductions for these concentrations in both growth media (Additional file 1: Table S2). For each strain background and condition, 4 independent founding populations were initiated (2× tagged with eGFP and 2× tagged with DsRED as fluorescent marker) resulting in 48 parallel *P. pastoris* cultures.

Initial growth profiling indicated marker protein (eGFP and DsRED) specific growth effects which were accounted for when calculating the competitive fitness and growth rates of evolved clones and populations (Additional file 1: Supplemental results and Additional file 1: Tables S2-S4). All populations were propagated by serial transfer for 500 generations; this equals a CCD of, on average, 10^10.38^ for each evolved population (Additional file 1: Table S5). Populations evolved on YPD and YPDN are denoted by YPD500 and YPDN500, whereas populations evolved on BMD and BMDN are denoted by BMD500 and BMDN500 hereafter.

### Growth characteristics and fitness of evolved *P. pastoris* populations

**Fig. 2:**
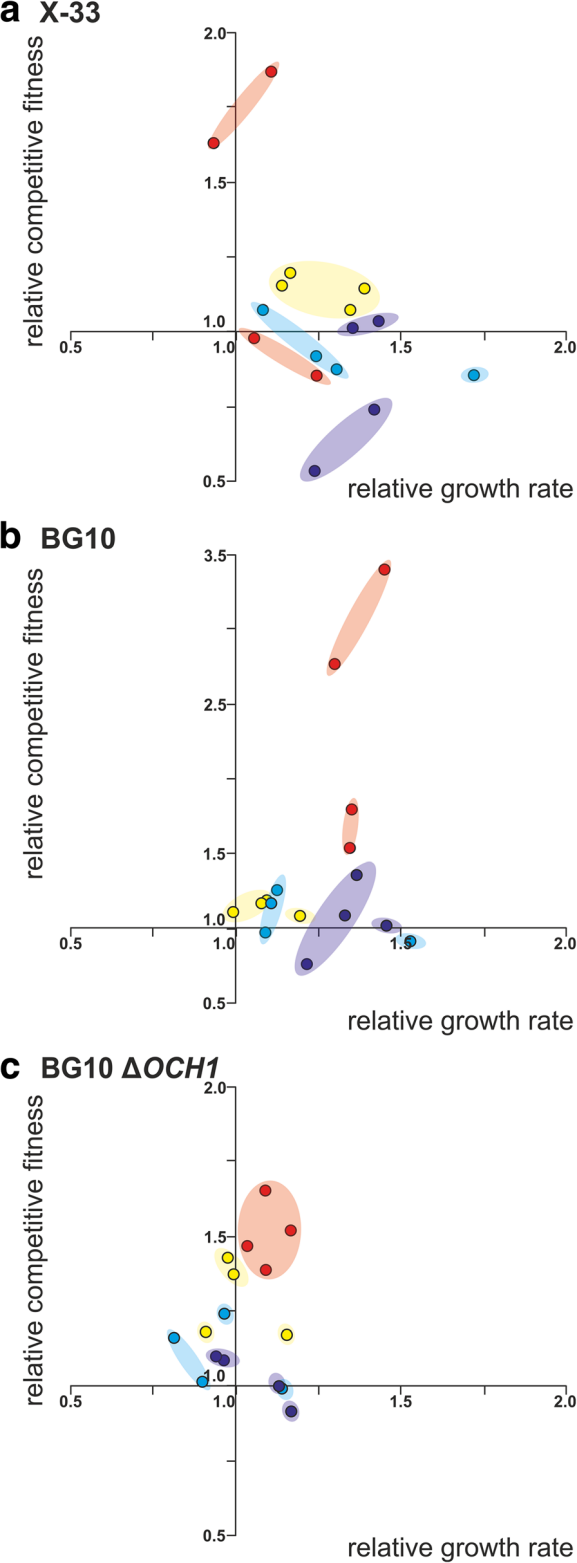
Correlation of growth rates and competitive fitness of evolved heterogeneous *P. pastoris* populations in adaptive conditions. Growth rate (x-axis) and competitive fitness (y-axis) relative to the ancestral strains. **a** *P. pastoris* X-33, (**b**) BG10 and (**c**) and BG10 ∆*OCH1*. Circles represent individual evolved populations adapted to YPD (yellow), YPDN (*red*), BMD (*light blue*) and BMDN (*dark blue*) growth conditions

The evolved wildtype populations were analyzed by means of maximum growth rate, as observed during exponential growth phase, and competitive fitness in comparison with the corresponding ancestral strains in the growth conditions used for long-term adaptation. For both parameters a high variation among the replicate populations for each growth condition was observed (Fig. 2). Although different fluorescent markers with an impact on the initial fitness were used (Additional file 1: Tables S2-S4), no marker protein specific bias of growth rate and fitness was identified. However, most of the evolved X-33 and BG10 wildtype populations showed increased growth rates after 500 generations of adaptation (Fig. 2a,b, Additional file 1: Table S6). Similarly, the evolved BG10 ∆*OCH1* populations showed high variation among the replicate populations and on average also lower growth rate improvements but more consistent competitive fitness increases (Fig. 2c). A common trend observed for populations of all genotype backgrounds were consistent fitness gains in YPD and YPDN rich medium conditions (Student’s t-test *p* ≤ 0.05 except for X-33 YPDN500 *p* = 0.22). In this context, significant growth rate increases across all replicate populations were only observed for the majority of evolved X-33 and BG10 wildtype backgrounds (Student’s t-test *p* ≤ 0.05 except for X-33 BMD500 *p* = 0.06). We selected the adapted X-33 populations for further pairwise evolved-evolved population competitions. YPD and YPDN-evolved populations showed mostly significant fitness advantages in comparison to minimal medium evolved populations in rich but also minimal growth conditions. Consistent with this observation, BMD- and BMDN-evolved populations did not show such advantages (Additional file 1: Fig. S2 and S3).

In many cases, the increase of growth rate was reported to come at the expense of biomass yield [44]. Our data indicate strain-specific differences (Additional file 1: Fig. S4a-c) but no clear correlation of growth rates and biomass yield among either evolved populations or single clones (Additional file 1: Fig. S4d).

Although the lag-phase of evolved populations was not analyzed in detail, in addition to the maximum growth rate during exponential phase, the growth rates of the early growth phase post-inoculation (first two hours) were analyzed. As compared to the ancestral strains, YPD and YPDN-adapted cultures showed on average 131% growth rate in adaptive conditions, whereas minimal medium-evolved cultures and cultures in non-adaptive conditions showed 89 and 99% of the ancestral growth rates in this early growth stage (Additional file 1: Table S7)*.* These data indicate that reduced lag-phases or increased growth rates after transfer from stationary can emerge in *P. pastoris* cultures evolved by serial transfer.

### Cross-benefits and trade-offs in non-evolutionary growth environments

**Fig. 3:**
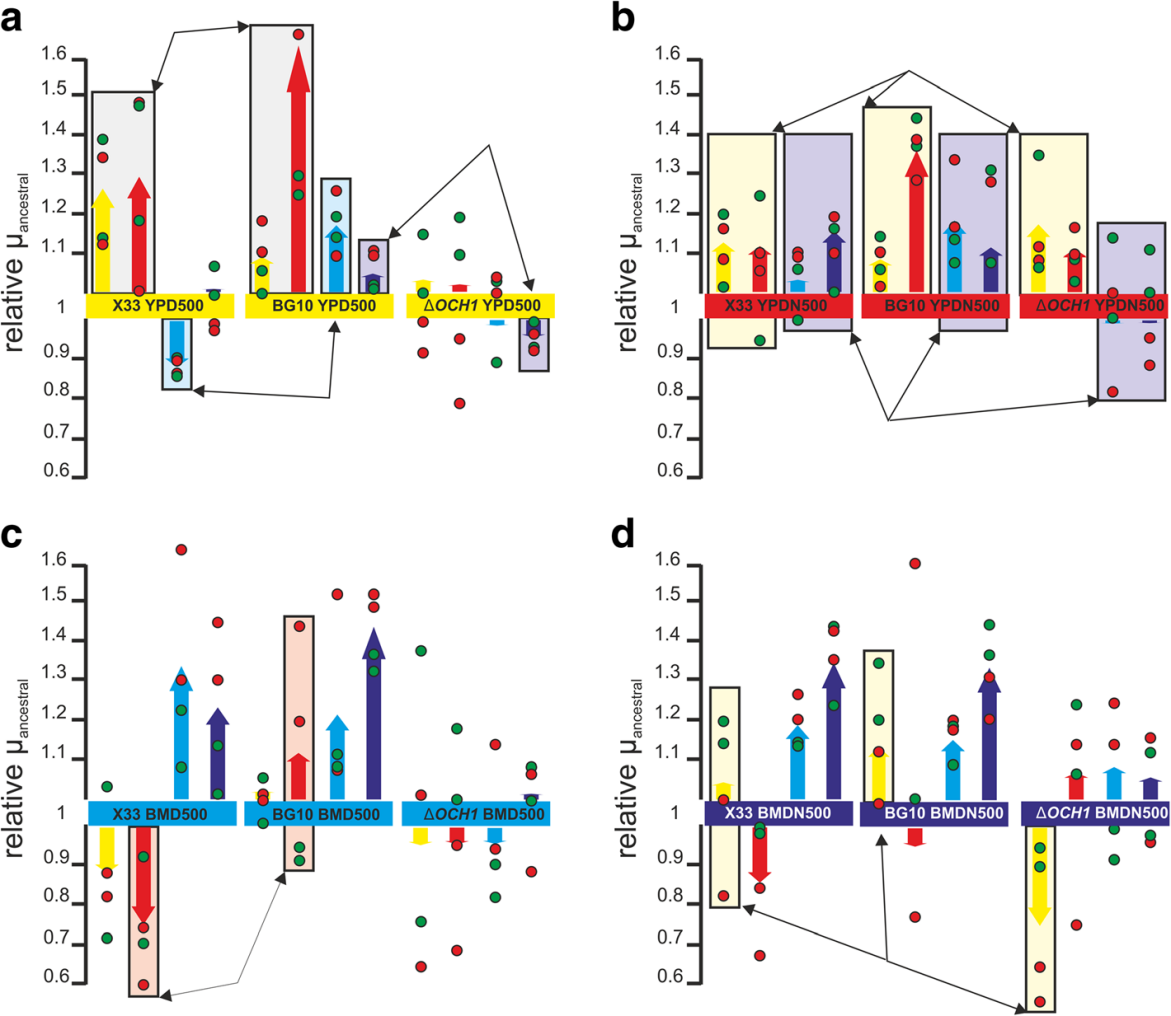
Growth rates of heterogeneous *P. pastoris* populations in non-evolutionary conditions. Growth rates for each genotype background are grouped based on the adaptive growth conditions (**a**) YPD, (**b**) YPDN, (**c**) BMD and (**d**) BMDN. Colored bars represent the average growth rates of all populations of each condition, whereas *dots* (*green* – eGFP populations, *red* – DsRED population) represent the growth rates of the individual populations. Growth rates: *yellow* – YPD, *red* – YPDN, *light blue* – BMD, *dark blue* – BMDN; important cross-benefit and trade-off correlations (either similar or contrasting) are highlighted in *boxes* and marked with *arrows*

Evolutionary trade-offs and cross-benefits in non-evolutionary conditions have been reported for previous laboratory evolution studies [2, 3, 45, 46]. Since growth rate represents a critical factor for biotechnological processes, we chose this parameter for further tests of all evolved populations across all growth conditions of this study (Fig. 3). Similar to the test performed in the adaptive conditions, the heterogeneous replicate populations showed a high degree of variation in non-evolutionary growth conditions but no specific bias due to the fluorescent marker protein (Fig. 3, Additional file 1: Table S6). For both wildtype strains, YPD-adaptation also resulted in a trend towards improved growth on YPDN. YPD-evolved BG10 populations showed a cross-benefit correlation (also improved growth) on BMDN, whereas the evolved BG10 ∆*OCH1* populations showed trade-offs (reduced growth) on BMDN (Fig. 3a). Independent of the genotypic background, YPDN-adapted populations showed largely cross-benefits on YPD (Fig. 3b). In both wildtype genetic backgrounds, but not in the Δ*OCH1* genetic background, YPDN-adaptation resulted in a clear trend towards cross-benefit correlation on minimal media (Fig. 3b). Furthermore, BMDN-adaption was highly compatible with growth on YPD in the wildtype populations, whereas a clear trade-off was observed in the *OCH1* knockout populations (Fig. 3d). Interestingly, we also observed diverging phenotypes in non-evolutionary conditions when comparing the wildtype backgrounds, as in the case of YPD-adapted populations during growth on BMD (Fig. 3a) and growth of BMD-adapted populations during growth on YPDN (Fig. 3c). The growth rate differences were also partially reflected by the competitive fitness in non-evolutionary conditions (Additional file 1: Fig. S5 and S6), but overall no clear correlation of maximal exponential growth rate and competitive fitness in the various growth environments was observed across all conditions tested within this study (Additional file 1: Fig. S7).

### Genome sequencing and mutational landscape of evolved *P. pastoris* clones

In order to establish a mutational landscape of the evolved *P. pastoris* populations, 3 random single clones were chosen from each evolved population and tested for their growth rates. The clones with the highest growth rates were selected for genome analysis by Illumina paired-end sequencing. In total 48 evolved *Pichia* genomes were sequenced: On average a 72-fold coverage was obtained for the X-33 clones and a 50-fold coverage for the BG10 and BG10 ∆*OCH1* clones (Additional file 1: Table S8).

91 mutations were identified in all 48 evolved clones (Additional file 1: Tables S9 – S11), resulting in ~1.75 mutations per clone. 61% of the total mutations represented single nucleotide polymorphisms (SNPs). C to T and G to A conversions were most frequent with 32 and 20%, respectively. Regarding insertion-deletion mutations, deletions were more frequent, representing 83% of all indel mutations (Additional file 1: Table S12). These data are in good agreement with previous results, indicating a high G:C to T:A prevalence of SNP mutations and a high frequency of deletions during experimental evolution [47].

The majority of the mutations (84%) were found in gene coding regions: 43% leading to amino acid conversions and 57% leading to premature stop codons in the corresponding gene products. Analysis of the open reading frames affected by such stop codons suggested that the majority of these mutations have an inactivating effect, leading to truncated gene products (Additional file 1: Fig. S8). About 13% of the mutations occurred in intergenic, non-protein coding regions. Two synonymous mutations, but no CNVs were identified.

**Fig. 4:**
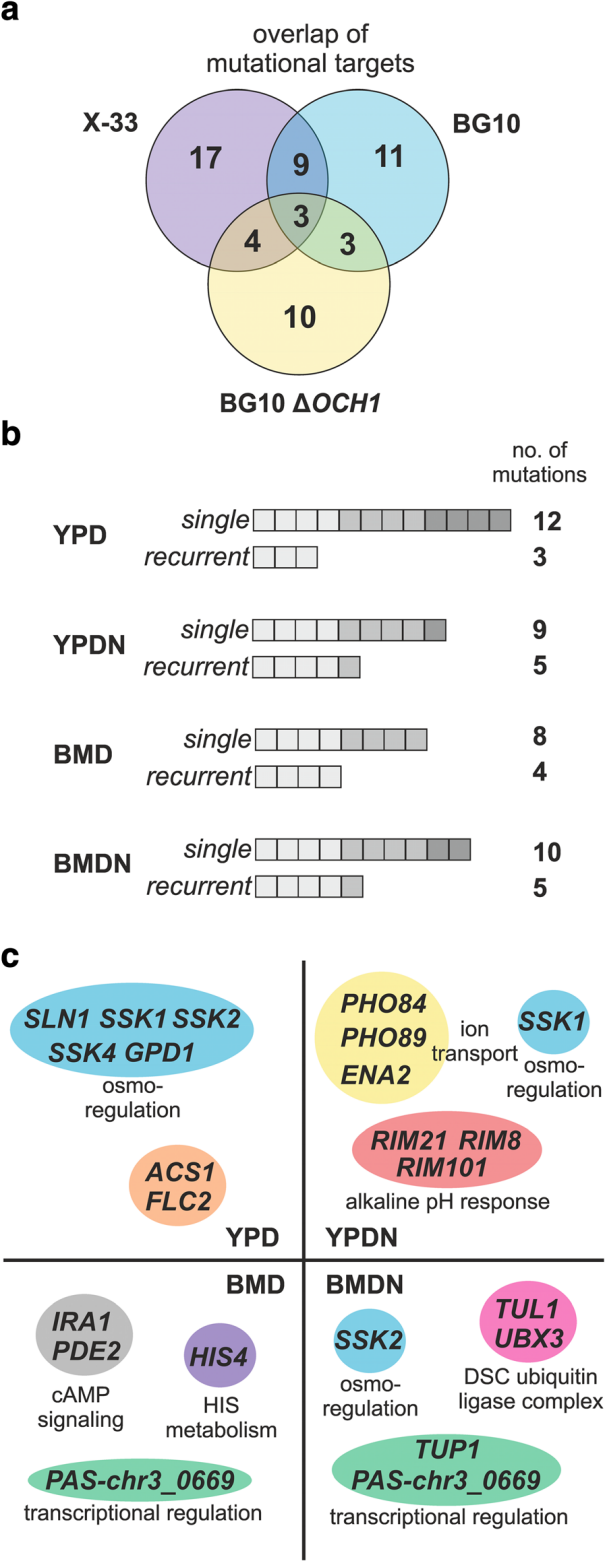
Mutations identified in evolved *P. pastoris* populations. **a** Overlap of mutational targets in the X-33 and BG10 wildtype and the BG10 ∆*OCH1* populations. **b** Number of single and recurrent mutations grouped by growth environment. **c** Environment-dependent mutations of genes and their associated functional modules. Only recurrently affected modules are shown

Generally, more mutational targets were observed for the evolved wildtype populations than for the ∆*OCH1* populations (28 and 20 for the wildtype clones and 14 total mutational targets for the 16 evolved ∆*OCH1* clones, respectively). Single mutations that only occurred in one of the evolved clones represented the majority of mutations (67%). With respect to convergent mutational targets among independently evolved clones, a higher overlap of mutational targets for the wildtype strains than for the wildtype vs. knockout combinations was observed (Fig. 4a). Gene ontology (GO) enrichment analysis using g:profiler [48] resulted in the identification of significantly enriched gene clusters (corrected *p*-value ≤0.05) related to: cellular response to abiotic stimulus (GO:0071214), cellular response to osmotic stress (GO:0071470), ubiquitin ligase complex (GO:0044695) and MAPK signaling (KEGG:04011). Grouped by growth condition, recurrent mutational targets were observed for all selective environments (Fig. 4b).

**Fig. 5:**
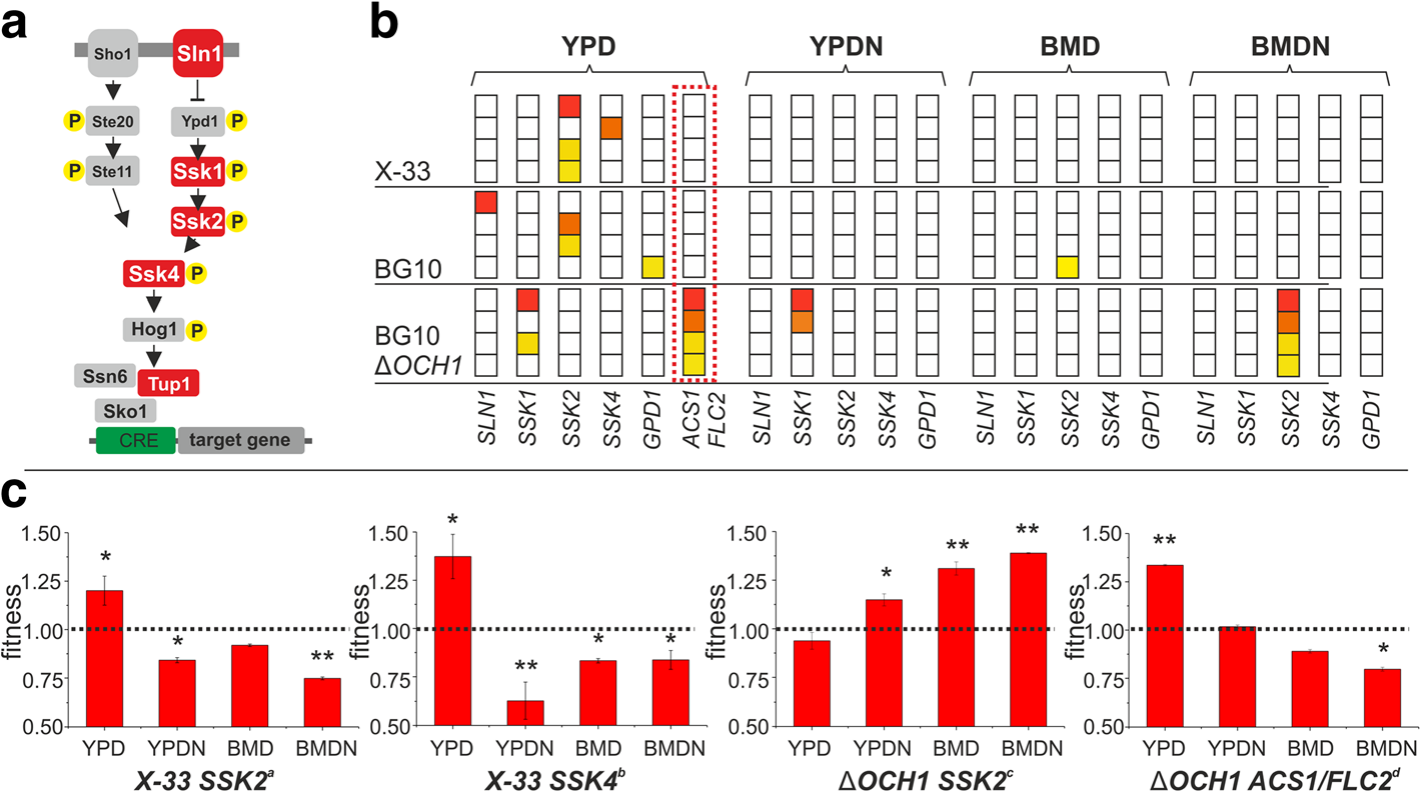
Osmotic stress-related adaptive mutations. **a** High osmolarity glycerol (HOG) MAPK signaling pathway (**b**) Mutations of HOG pathway related genes in the evolved *P. pastoris* clones isolated from independent populations. Mutations are grouped by their appearance in strain background and growth condition. For each growth condition the affected genes are shown. The four clones for each condition are represented by squares. The occurrence of a particular mutation is indicated by color (*yellow* to *red* for the presence of a mutation in each individual clone). A *blank square* indicates the absence of a mutation. Non-HOG-related mutations (*ACS1/FLC2*) in ∆*OCH1* clones are highlighted by a *dashed box*. (**c**) Fitness of clones with a single HOG-related or *ACS1*/*FLC2*-related mutation (**a**-**d**, as described in Tables S13-S15). Values represent averages +/− standard deviation of *n* = 4; paired Student’s T-test values * *p* ≤ 0.05, ** *p* ≤ 0.01

**Fig. 6:**
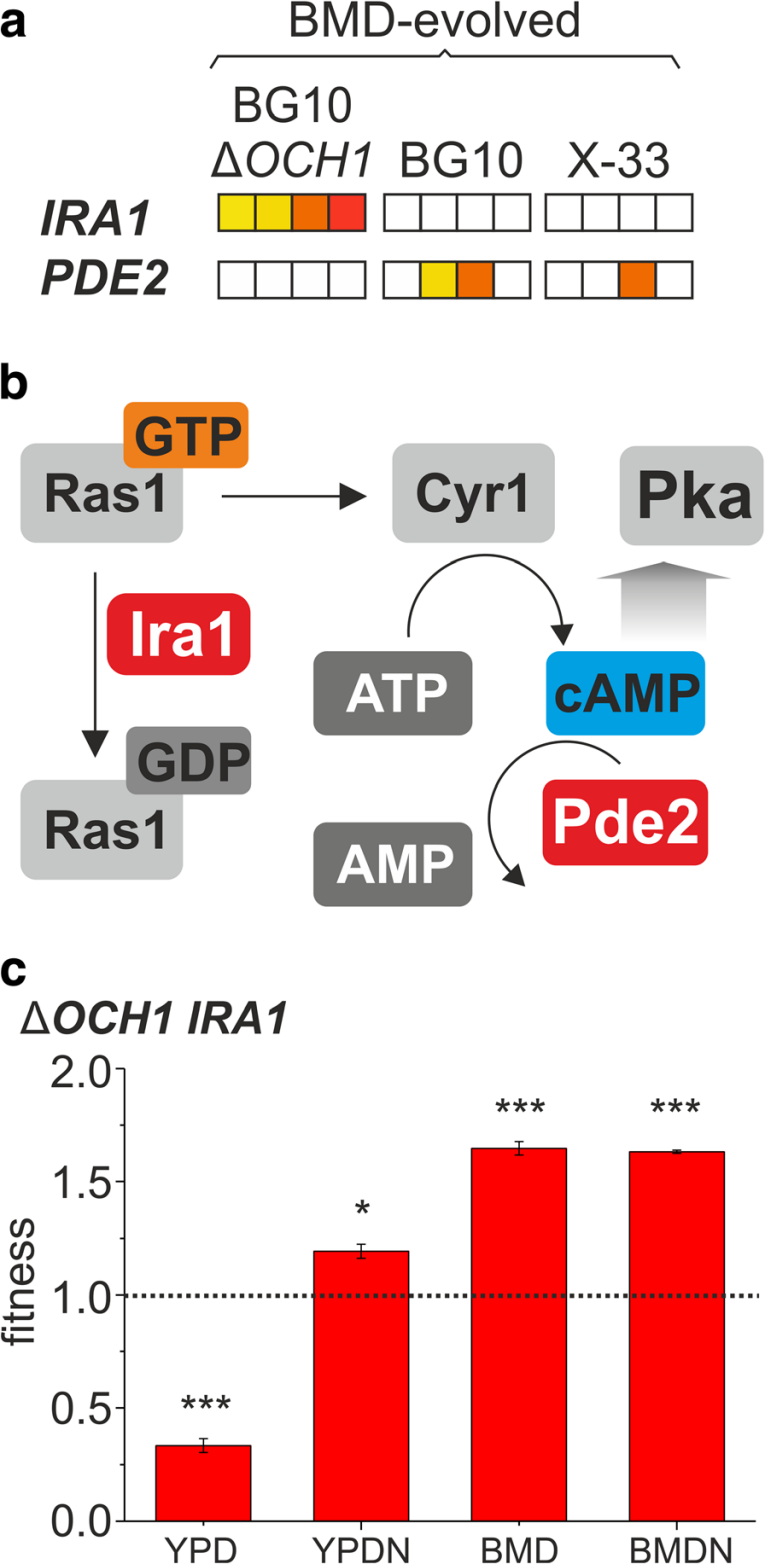
cAMP/PKA signaling mutations in *P. pastoris* clones adapted to minimal growth medium (BMD). **a** Occurrence of mutations grouped by strain background. The occurrence of a particular mutation is indicated by color (*yellow* to *red* for the presence of a mutation in each individual clone). A *blank square* indicates the absence of a mutation. **b** Enzymatic steps catalyzed by the mutated genes *IRA1* and *PDE2.* **c** Fitness effect of a single *IRA1* mutation in an evolved *OCH1* clone (clone *∆OCH1* BMD G2b Tables S13-S15). Values represent averages +/− standard deviation; *n* = 4; paired Student’s T-test values * *p* ≤ 0.05, ** *p* ≤ 0.01, *** *p* ≤ 0.001

Convergent evolution at mutational hotspots is frequently observed on the gene- or functional module level [49]. Here we observed a very high degree of environment-dependent convergence with a bias towards cellular signaling, transcriptional regulation, pH response and ion transport (Fig. 4c). Additionally, specific recurrent targets appeared to be highly genotype-dependent since they were only identified in individual genetic backgrounds (Fig. 5, Fig. 6 and Additional file 1: Fig. S9).

### HOG-pathway mutations

The majority of the identified recurring mutations were related to the high osmolarity glycerol (HOG) signaling pathway (Fig. 5a) and were mostly associated with YPD-adaptation, since in 83% of all YPD-evolved clones HOG-pathway related mutations were found (Fig. 5, Additional file 1: Tables S9-S11). All YPD-evolved X-33 and BG10 wildtype clones harbored a mutation in genes related to osmotic stress response mechanisms. Seven out of eight mutations were related to the *SLN1*-branch of osmotic stress signaling, affecting *SSK2/4* and *SLN1*. These mutations predominantly led to loss-of-protein-function mutations, thereby inactivating the *SLN1* signaling cascade (Additional file 1: Fig. S8). Two HOG-related *SSK1* mutations were also identified in YPD-evolved *OCH1* knockout clones. However, in 50% of the clones with a wildtype background, HOG mutations represented the only mutations that were identified, whereas in ∆*OCH1* clones a different mutational hotspot (*ACS1*/*FLC2* locus) was predominant. In clones adapted to YPDN and BMD, only 3 out of 12 clones had such a mutation (Fig. 5b). Interestingly, whereas in none of the sequenced salt stress-adapted clones with wildtype genetic background a HOG-related mutation was identified, all BMDN-adapted BG10 ∆*OCH1* clones and 2 YPDN-adapted clones showed mutations of either *SSK1* or *SSK2* (Fig. 5b).

To analyze the specific effect of these HOG-related mutations, *P. pastoris* clones, for which only a single mutation related to the *SSK2* and *SSK4* gene was identified, were chosen for extended growth profiling. The two chosen X-33 wildtype clones (X-33 YPD GFP 1a and GFP 2b) showed similar fitness profiles with a clear advantage on YPD and fitness loss in the remainder conditions as compared to the ancestral strains (Fig. 5c). In contrast, a BMDN-evolved ∆*OCH1* clone with a single *SSK2* mutation showed no growth advantage on YPD but a fitness gain on YPDN and both BMD-based growth media (Fig. 5c). Similar results were obtained for growth rates and biomass yields (Additional file 1: Table S13 and S14). An additional series of viability assays with these clones upon osmotic shock treatment (4 M NaCl, 2 h) and heat shock treatment (50 °C, 30 min) showed that HOG-related mutations did not result in decreased viability upon shock treatment as compared to the ancestral strains, neither in the wildtype nor in the ∆*OCH1* strain background (data not shown).

### *ACS1/FLC2* locus

Each of the YPD-evolved ∆*OCH1* clones had a mutation in the upstream region between the open reading frames for *ACS1* and a *FLC2*-like gene. These intergenic mutations resided closer to the *FLC2*-like open reading frame (Additional file 1: Fig. S8) but could potentially affect the expression of both genes. qPCR analysis revealed that in all YPD-evolved clones the expression of the *FLC2* gene was reduced (Additional file 1: Fig. S10a), whereas no consistent trend was observed for *ACS1* expression (Additional file 1: Fig. S10b). Growth profiling of such a clone with a single mutation in this intergenic region between the two genes revealed that it had a clear fitness and growth advantage on YPD and a growth trade-off pattern in the remainder conditions similar to HOG-related mutations in the wildtype genetic background (Fig. 5c). Overexpression of both genes in the ancestral backgrounds was performed but except for reduced growth rates in all growth conditions upon overexpression of *ACS1* in the ancestral *OCH1* strain and improved growth in BMD for the wildtype strains, no comprehensive impact was observed (Additional file 1: Fig. S10c-f).

### A novel *P. pastoris*-specific transcription factor as convergent mutational target

Recurrent mutations were also found in the hitherto uncharacterized open reading frame PAS-chr3_0669 in BMD- and BMDN-evolved wildtype clones (Additional file 1: Fig. S9). PAS-chr3_0669 encodes a *GAL4*-like zinc finger transcriptional regulator of the MHR (middle homology region) superfamily. Growth profiling an evolved clone with a likely singular mutation in this open reading frame (X-33 BMD GFP 1a) showed increased fitness in minimal growth media and growth trade-offs in YPDN growth medium (Additional file 1: Tables S13-S15).

**Fig. 7:**
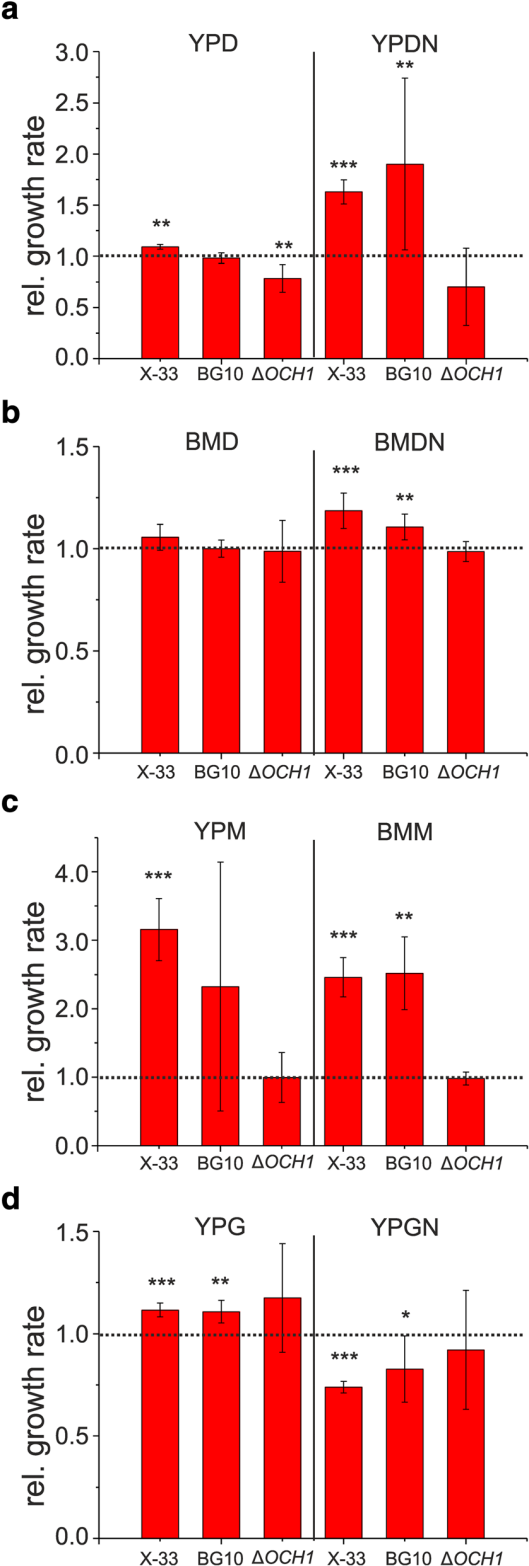
Effect of PAS-chr3_0669 overexpression in the ancestral strains. The effect of overexpression of the *GAL4*-like gene in comparison to an empty vector strain is shown for all ancestral strains in (**a**) YPD and YPDN rich media (**b**) BMD and BMDN minimal media (**c**) rich and minimal methanol growth media, YPM and BMM and (**d**) rich glycerol growth media, YPG and YPGN. Values represent averages +/− standard deviation; *n* = 4; paired Student’s T-test values * *p* ≤ 0.05, ** *p* ≤ 0.01, *** *p* ≤ 0.001

Gal4-like transcription factors are ubiquitous in fungi and are involved in the regulation of a plethora of cellular processes, including metabolic processes and stress response [50]. However, the specific function of PAS-chr3_0669 has not been elucidated. Thus, we performed an assessment of the implications of this transcriptional regulator by overexpressing the gene in the ancestral genetic backgrounds. Overexpression conferred a growth rate improvement in glucose-based rich and minimal media when salt stress was applied (Fig. 7a,b). Considering the importance of other carbon sources, such as glycerol and methanol, during *P. pastoris* bioprocesses [20], these carbon sources were also tested. Growth on methanol as carbon source led to significantly increased growth rates in both, rich and minimal growth media (Fig. 7c), whereas an inverse effect for growth on salt stress medium was observed when glycerol was used as carbon source (Fig. 7d). Altogether these data show that this *P. pastoris-*specific transcriptional regulator has a multifaceted role in stress and carbon source signaling.

### Differential sets of mutations associated with salt stress adaptation

Salt stress adaptation let to the accumulation of environmentally different mutational targets. In the wildtype populations, BMDN-adaptation led to mutations of PAS-chr3_0669 and the transcriptional repressor *TUP1*, with *TUP1* mutations present in both genetic wildtype backgrounds. As discussed above, all BMDN-evolved ∆*OCH1* clones had mutations in the *SSK2* MAPKKK involved in osmotic stress response (Fig. 5b and Additional file 1: Fig. S9).

In contrast, a higher convergence of mutational targets was observed for YPDN-evolved clones: Phosphate transporter (*PHO84* and *PHO89*) mutations were only identified in the wildtype genetic backgrounds, but mutations of the sodium transporter *ENA2* and components of the pH stress response (Rim*)* pathway were identified in all genetic backgrounds (Additional file 1: Fig. S9).

The sodium pump *ENA2* is essential for salt tolerance [51] and overexpression of the *ENA* gene cluster has been shown to be an adaptive strategy during salt stress in evolving *S. cerevisiae* populations [52]. Several functionally distinct *ENA* isoforms are known in *S. cerevisiae* [53]*,* whereas genome data suggest only one isoform in *P. pastoris*. The mutations leading to amino acid conversions as observed in the current study (Additional file 1: Tables S9-S11) could therefore alter substrate preferences to specifically facilitate sodium efflux at the cost of decreased affinity towards other monovalent metal ions.

Potential implications of *PHO* gene and Rim-pathway mutations are less clear. YPDN-adaptation was performed at a pH of 7.4 and could indicate adaptation towards mildly alkaline conditions, since it has been reported that alkalinization mimics phosphate starvation conditions and induces the expression of genes related to phosphate uptake in *S. cerevisiae* [54]. Similarly, the Rim pathway is induced during alkaline pH stress in *S. cerevisiae* and is also known to involve the induction of *ENA* expression [55]. Therefore, Rim pathway mutations could indicate an alternative route to increase sodium efflux in *P. pastoris* due to the absence of multiple *ENA* isoforms.

## Discussion

### General properties of evolving *P. pastoris* populations

The fitness of the adapted *P. pastoris* populations that were observed in this study (environment-dependent ω_mean_ ranging from ~1.02 to 1.50) and the observed growth rates (μ_average_ in adaptive conditions of 118%) are well within the order of magnitude that was observed for other microbial species selected by serial transfers or in nutrient-limited chemostat cultures [56–58]. Furthermore, the generally low correlation of fitness and exponential growth rates (Additional file 1: Fig. S7) indicated additional factors that contribute to population fitness. In this context, increased growth rates in early (lag-phase) stages of batch cultures, as observed in certain populations in the current study (Additional file 1: Table S7), can contribute the overall fitness observed for environmentally-selected *P. pastoris* populations. However, single clone growth profiling also indicated substantial population heterogeneity after 500 generations of selective growth. Heterogeneity is regularly observed in experimental evolution studies [59] and can even represent a selected trait, particularly in stress-adapting yeast populations [60].

In serial transfer experiments, beneficial mutations can be also routinely lost to drift [47] and genome analysis of a single clone from each population, as performed in the current study, does not represent the full inter-population mutational landscape. Nevertheless, the high degree of convergence among mutational targets indicated an extensive degree of directional selection (Fig. 4, Fig. 5, Fig. 6 and Additional file 1: Fig. S9) among independent growth rate-selected *P. pastoris* cells. Furthermore, the number of mutations identified for each clone (ranging from 1 to 5 mutations) is in accordance with previous experiments using asexually propagated microbial cultures [61]. Although the expression of the marker protein resulted in fitness differences among the ancestral strains (Additional file 1: Supplemental results and Additional file 1: Tables S2-S4), no reporter protein-specific bias was observed, suggesting either a minor or similar evolutionary cost of low-level eGFP and DsRED expression. As expected, largely parallel adaptive trends were observed for the wildtype populations (X-33 and BG10). Nevertheless, recurrent *HIS4* mutations were only identified in BMD-adapted X-33 clones (Additional file 1: Fig. S9, Tables S9-S11). In agreement with the absence of *HIS4* mutations in the BG10 genetic background, genotype-dependent phenotypic differences were observed for the ancestral (Additional file 1: Table S2) and evolved cells (Fig. 3a,c) indicating uncharacterized genetically-linked differences among prototrophic *P. pastoris* laboratory strains.

### Mutations related to transcriptional regulation

Changes to cellular regulatory hubs can have a great impact on the gene expression state of a microbial cell. Therefore, mutations of such hubs are frequent during experimental evolution [62–64].

Most notably, BMD and BMDN-adapted wildtype clones showed mutations in the *P. pastoris-*specific Gal4-like transcriptional regulator, which is involved in environmental sensing (Fig. 4c, Fig. 7 and Additional file 1: Fig. S9). Similarly, mutations of the transcriptional repressor *TUP1* were identified in both wildtype backgrounds adapted to BMD and BMDN media (Additional file 1: Fig. S9). There is little information about the exact implications of Tup1 in the transcriptional control of *P. pastoris.* In line with mutations identified upon minimal growth media adaption, *TUP1* overexpression in the ancestral strains indicated gene dosage effects on growth rates, particularly in minimal and stress media conditions but also during growth on methanol as carbon source, indicating the involvement of Tup1 in the regulation of nutritional competence, stress and methanol metabolism (Additional file 1: Fig. S11). Further mutations were identified in the genes *IRA1* and *PDE2* (Fig. 6a), with their gene products involved in the regulation of cellular cAMP levels (Fig. 6b). Among cellular regulatory cascades, the cAMP / protein kinase A (Pka) pathway plays a major role in growth signal integration by promoting growth and repressing stress response signals upon glucose availability in *S. cerevisiae* [65]. In the current study, growth characterization of a clone with a singular *IRA1* mutation indicated that this mutation conferred a fitness advantage during growth on minimal media and salt stress at the expense of growth on YPD (Fig. 6c, Additional file 1: Tables S13-S15).

Previous studies also identified mutations of Gal4-like transcription factors and *TUP1* to be associated improved growth of *P. pastoris*. [25, 27]; therefore these regulatory hubs and the cAMP/Pka pathway could represent interesting engineering targets for growth optimization of recombinant *P. pastoris* host strains, specifically in minimal growth conditions.

### Importance of HOG-signaling loss-of-function mutations

A major finding in this study is the highly convergent selection of HOG-signaling mutations (Fig. 5b). The HOG pathway (Fig. 5a) plays a crucial role in the response to osmotic stress conditions [66]. The osmotic stress response is also highly interconnected with other cellular stress response mechanisms such as the response to cold and oxidative stress [67, 68] and with nutrient signaling pathways in baker’s yeast; stress protection and growth competence are tightly balanced in microbial cells [69, 70]. We show that the loss of osmotic stress signaling leads to a growth promoting effect in nutrient rich (YPD) conditions (Fig. 5c, Additional file 1: Tables S13-S15). Additionally, in a smaller study with the same basic growth conditions but with methanol as carbon, selection on nutrient rich YP medium also led to the identification of an evolved clone with a HOG-signaling (*SLN1*) mutation with similar growth effects on YP-based and BM-based growth media [27]. These results are not unlike the effects during environmental adaptation in *S. cerevisiae*. A high frequency of HOG-signaling inactivating mutations was previously observed during nutrient-limited (chemostat) selection [64]. However, it has to be noted that the long-term propagation of microbial cultures by serial transfer represents a substantially different growth environment, since the populations were not strictly limited in carbon supply but experienced lag-, exponential- and stationary phase for each growth cycle. Thus, the general loss of stress signaling capacity represents an even more important evolutionary strategy for adapting yeast populations than previously anticipated.

In this context, it is noteworthy that HOG-signaling mutations occurred in 6 out of 8 sequenced ∆*OCH1* clones evolved under salt stress and, in contrast to the evolved wildtype clones, conferred a fitness advantage in salt stress conditions (Fig. 5b and c). Och1 has been shown to be a Golgi-resident enzyme [71] but its deletion causes the up-regulation of genes involved in endoplasmic reticulum (ER) protein folding, ER-associated protein degradation and protein trafficking in an *HAC1*-independent manner in *S. cerevisiae* [72]. Glycosylation deficiency also leads to defects of fungal cell wall integrity [24, 73], thereby triggering the unfolded protein response (UPR) as shown for *Aspergillus fumigatus* [73] and *S. cerevisiae* [74]. Moreover, it was shown that in *P. pastoris* salt stress induces a UPR-like response (e.g. up-regulation of Ssa4, Ssb1 and Pdi1). However, this effect was diminished in recombinant protein-expressing cells, most likely due to protein folding stress-related pre-conditioning [43]. Thus, there is evidence that protein glycosylation-related and osmotic stress signals converge at the ER/Golgi complex. The augmentation of the UPR through both routes, caused by an *OCH1* gene deletion and the HOG pathway, could lead to a ‘runaway’ stress response, diminishing growth proliferating signals detrimental towards improved growth of ∆*OCH1* cells during salt stress exposure. However, in contrast to the wildtype background, the loss of HOG-signaling does not improve growth in YPD and led to a strong selection of convergent mutations in the *ACS1/FLC2* in order to provide a fitness landscape comparable to the HOG-related mutations in YPD-evolved wildtype cells (Fig. 5c). Interestingly, in *S. cerevisiae FLC2* is involved in maintaining cell wall integrity [75]. Mutants displayed loss of cell wall mannose phosphates and Flc2 has been proposed to be essential for ER FAD uptake and disulfide bond formation. Furthermore, several mannosyltransferases have been described as high copy suppressors of synthetic lethality of *FLC* gene deletions [75]. Thus, this genetic locus represents an interesting target for further experimentation, especially in the context of glyco-engineered *P. pastoris* strains.

### Implications of glycosylation-deficiency

The *OCH1* gene product is essential in the N-glycosylation machinery of *P. pastoris* (Fig. 1b), and node removal by gene knockout is biotechnologically important for the production of recombinant proteins with small- or humanized N-glycans. Previous research indicated a broad capability of microbial populations to develop compensatory mutations upon node-removal [17, 18].

On the population level wildtype cells showed on average 25% growth rate increase whereas *OCH1* populations showed only a ~ 3.5% increase. Particularly in stressful YPDN and BMDN conditions, *OCH1* populations showed significantly lower (Student’s T-test *p* ≤ 0.05) growth rate improvements as compared with otherwise isogenic BG10 populations. Thus, similar to *rpoS* node removal in *E. coli* [16], *OCH1* deletion resulted in an environment-dependent reduction of the adaptive potential.

We also observed fewer and highly selective mutational targets in the adapted ∆*OCH1* clones (Fig. 4a, Additional file 1: Table S11). Single clone growth profiles (e.g. YPD- and BMDN-evolved ∆*OCH1* cells) showed that the identified mutations are major contributors to growth behavior observed on the population level (Fig. 3, Fig. 5 and Fig. 6). The largely different genetic mutations suggest diverging evolutionary trajectories of evolving *OCH1* populations; the most important example being the environment-dependent emergence and effect of HOG-related (*SSK2)* mutations in the wildtype and *OCH1* populations (Fig. 5). Additionally, recurrent targets (including PAS-chr3_0669 and *TUP1*) were observed in the wildtype but not in the *OCH1* genetic background (Fig. 5, Fig. 7 and Additional file 1: Fig. S9). Even simple gene dosage increases of PAS-chr3_0669 and *TUP1* resulted in significantly different growth rate effects in the ancestral wildtype and ∆*OCH1* strains (Fig. 7 and Additional file 1: Fig. S11). Considering the relatively small sample size of the current study, this observation is conditionally but points toward a high degree of incompatibility of the evolutionary solutions among wildtype and glycosylation-deficient clones.

On a broader scale, these data show how artificial or natural mutations can play a role in the early steps of speciation, even in non-diverging growth environments. From a biotechnological perspective this incompatibility indicates the need for specific engineering strategies of glyco-engineered *P. pastoris* strains.

### Adaptive solutions in comparison to the model yeast *S. cerevisiae*

Despite the principal conversation of cellular regulation, the regulatory pathways can vary even among closely related species due to niche-specific requirements [12]. *P. pastoris*, first isolated from tree resin, occupies a different ecological niche than *S. cerevisiae* and has different metabolic capabilities: *P. pastoris* can grow on methanol as sole carbon source [39]. Furthermore, *S. cerevisiae* is a Crabtree-positive yeast species whereas *P. pastoris* belongs to the Crabtree-negative clade, showing minimal ethanol production during aerobic growth on excess glucose. The Crabtree-positive growth phenotype was estimated to have occurred around 125–150 mya, coinciding with the development of the first fruit plants [76]. *P. pastoris* does not possess the expanded hexose transporter (*HXT*) gene cluster of *S. cerevisiae* [77], which is suggested to be one of the prerequisites for Crabtree (overflow) metabolism.

We identified several mutational targets that were previously observed in similar studies with *S. cerevisiae*, including genes such as *ENA1/2*, *IRA1/2, PDE2, SSK1/2* and *TUP1* [64, 78]. In contrast to many *S. cerevisiae* studies, no hexose metabolism related mutations were found [46, 64, 79]. This may be in part due to the serial transfer selection strategy, but *P. pastoris* is also missing a high similarity homologue of the important repressor Rgt1, suggesting differences of metabolic control in agreement with different lifestyles. In this context, the adaptation to glucose did not lead to significant growth trade-offs during the growth on glycerol, an important carbon source for biomass accumulation in *P. pastoris* fed batch processes. On the contrary, we observed that glucose adaptation resulted in growth improvements on glycerol, particularly for populations adapted to minimal growth media (Additional file 1: Fig. S12). This is in agreement with previous results that showed similar transcriptional patterns of *P. pastoris* during growth on glucose and glycerol [80].

## Conclusion

Our data show that optimization by the mutation of conserved key regulatory components of stress and nutrient signaling represents a universal feature during experimental evolution in *P. pastoris* and other yeasts such as the model yeast *S. cerevisiae*. However, in conjunction with previous results we also provide evidence for highly species-and genotype-specific mutations, which improves our understanding of the regulatory features of wildtype and mutant strains of biotechnologically relevant yeast species.

## Additional file

Additional file 1: Contains Supplemental Results, **Tables S1–15** and **Figures S1–12**. (PDF 1490 kb)

## Abbreviations

BMD: buffered minimal dextrose medium
cAMP/Pka: cyclic AMP / Protein kinase A
CCD: cumulative number of cell divisions
CNV: copy number variation
DsRED: *Discosoma sp*. red fluorescent protein
eGFP: enhanced green fluorescent protein
ER: endoplasmic reticulum
FAD: Flavin adenine dinucleotide
GO: gene ontology
HOG: high osmolarity glycerol
*HXT*: hexose transporter
Indel: insertion/deletion
KEGG: Kyoto Encyclopedia of Genes and Genomes
*m*: realized Malthusian parameter
MAPK: mitogen-activated protein kinase
*OCH1*: outer chain elongation factor 1
OD_600_: optical density at 600 nm
qPCR: quantitative polymerase chain reaction
SNP: single nucleotide polymorphism
UPR: unfolded protein response
YNB: yeast nitrogen base
YPD: yeast extract peptone dextrose complete medium
ω: relative fitness
