# Supplementary material for: The adaptive landscape of wildtype and glycosylation-deficient populations of the industrial yeast Pichia pastoris
Source: BMC Genomics. 2017 Aug 10;18:597. doi: 10.1186/s12864-017-3952-7 (PMC5553748; doi:10.1186/s12864-017-3952-7)
Supplement: Additional file 1: — Contains Supplemental Results, Tables S1–15 and Figures S1–12. (PDF 1490 kb) [file 12864_2017_3952_MOESM1_ESM.pdf]

The adaptive landscape of wildtype and glycosylation-deficient populations of the industrial yeast *Pichia pastoris*

Josef W. Moser[1,2], Iain B.H. Wilson[1] and Martin Dragosits[1*]

[1] Department of Chemistry, University of Natural Resources and Life Sciences, Muthgasse 18, 1190 Vienna, Austria

[2] Austrian Centre of Industrial Biotechnology (ACIB), Muthgasse 11, 1190 Vienna, Austria

josef.moser@acib.at
iain.wilson@boku.ac.at
martin.dragosits@boku.ac.at

Additional File 1

***Corresponding author:**
Martin Dragosits, University of Natural Resources and Life Sciences, Vienna, Department of Chemistry. Muthgasse 11, A-1190 Vienna; Tel. +43 1 47654 77222, e-mail: martin.dragosits@boku.ac.at

Supplemental Results

Growth characteristics of ancestral *P. pastoris* strains

For the purpose of subsequent direct competition assays, *P. pastoris* strains were tagged by the expression two recombinant reporter proteins. The *PGK1* (phosphoglycerate kinase) promoter (P_{PGK1}) was applied for fluorescent protein expression (Figure S1). While this promoter is active over a wide range of growth conditions, it results in low recombinant protein expression as compared with other promoters [1]. In this context, metabolic engineering and recombinant protein production have been reported to pose a metabolic burden on microbial host cells [2]. Specifically, in a recent study even moderate recombinant protein production resulted in a detectable metabolic burden in *P. pastoris* [3]. Thus, we analyzed the growth characteristics and fitness of the founding strains in detail.

Subtle growth rate differences between strains BG10 and X-33 on BMD medium indicated minor differences between the ancestral wildtype strains. Furthermore, the DsRed-tagged X-33 strain showed a decreased growth rate on YPDN as compared with the corresponding GFP-tagged strain (Table S2). Similarly, direct competition assays indicated decreased fitness (ω) of DsRed-expressing wildtype strains across all growth conditions (Table S3). Thus, a metabolic burden due to marker protein expression was observed.

In contrast to the ancestral wildtype strains, marker protein-dependent fitness differences were either absent or even reversed in the ancestral BG10 Δ*OCH1* strains (Table S3). Potential differences between the BG10 wildtype and BG10 Δ*OCH1* strain were analyzed. The wildtype only showed a clear growth rate advantage over the GFP- or DsRED-tagged Δ*OCH1* strains on YPD but not on salt stress and minimal growth media (Table S2). Additional competition assays of ancestral BG10 and Δ*OCH1* strains revealed a significant effect of the recombinant protein marker itself; e.g. the DsRed-expressing wildtype strain showed increased competitive fitness over the eGFP-expressing *OCH1* knockout strain during growth on YPD, whereas the *vice versa* combination showed nearly identical fitness (Table S4).

Although the initial fitness differences had to be considered in the calculation of competitive fitness of the evolved populations, it also offered the opportunity to analyze a potential bias during laboratory evolution due to non-neutral markers.

Whereas possible explanations for these phenomena include copy number variations and differences in the amino acid composition of the fluorescent proteins, these data also indicate a more complex correlation of N-glycosylation deficiency, recombinant protein burden and the environmental growth context than previously reported [4, 5] and will merit a detailed analysis in a separate study.

Supplemental Tables

name	5' → 3' sequence	purpose
Ppgk1__BglII_fw	AAAGATCTAAGTTGGTACCCAGCCGATCAC	reporter plasmid
pPpgk1_EcoRI_rv	ATGAATTCTTTCGTAATCAATTGGGCTATG	reporter plasmid
eGFP_EcoRI_fw	ATGAATTCATGGTGAGCAAGGGCGAGGA	reporter plasmid
eGFP_NotI_rv	ATGCGGCCGCTTACTTGTACAGCTCGTCCAT	reporter plasmid
DsRed_EcoRI_fw	ATGAATTCATGGCCTCCTCCGAGAACGT	reporter plasmid
DsRed_NotI_rv	ATGCGGCCGCTTACAGGAACAGGTGGTGGC	reporter plasmid
ACS1_fw	ACAACTATTTCGAAACGAGGAATTCATGCCATTAGATAACGAACA	cloning pGAPzB
ACS1_rv	GTTTTTGTTCTAGAAAGCTGGCGGCCGCTCATTTGCGGGCATCCCTTT	cloning pGAPzB
chr3_0669_fw	ACAACTATTTCGAAACGAGGAATTCATGCCGAAACGTAATAAGAC	cloning pGAPzB
chr3_0669_rv	GTTTTTGTTCTAGAAAGCTGGCGGCCGCTCAGCCTCTGTGGAAGTTGA	cloning pGAPzB
FLC2_fw	ACAACTATTTCGAAACGAGGAATTCATGACCATAGTGACGTGTGC	cloning pGAPzB
FLC2_rv	GTTTTTGTTCTAGAAAGCTGGCGGCCGCCTAGTTCCATCTAACGTCTT	cloning pGAPzB
TUP1_fw	ACAACTATTTCGAAACGAGGAATTCATGTCGTACAACAGACCATT	cloning pGAPzB
TUP1_rv	GTTTTTGTTCTAGAAAGCTGGCGGCCGCTCAAGTCCACTTCCAGATTC	cloning pGAPzB
ACS1_qPCR_fw	GAATCCAGACAAACCTGCC	real-time PCR
ACS1_qPCR_rv	GTCACCCTTTTTCACACCC	real-time PCR
FLC2_qPCR_fw	ACATCCGAAAAATACCACAGAC	real-time PCR
FLC2_qPCR_rv	CCATAGAACCTTCCATAGCAC	real-time PCR
ACT1_qPCR_fw	CCAAACCACTTACAACTCCATC	real-time PCR
ACT1_qPCR_rv	GCATACGCTCAGCAATACC	real-time PCR

Table S1. DNA oligonucleotides used in the current study. List of oligonucleotides that were used for cloning procedures and real-time PCR.

	strain	
	**X-33**	
**growth medium**	eGFP	DsRed
YPD	0.322 +/- 0.010	0.323 +/- 0.005
YPDN	0.283 +/- 0.006	0.233 +/- 0.021
BMD	0.321 +/- 0.002	0.326 +/- 0.022
BMDN	0.252 +/- 0.003	0.256 +/- 0.010
	**BG10**	
**growth medium**	eGFP	DsRed
YPD	0.292 +/- 0.013	0.289 +/- 0.021
YPDN	0.199 +/- 0.007	0.209 +/- 0.005
BMD	0.231 +/- 0.010	0.223 +/- 0.010
BMDN	0.225 +/- 0.006	0.213 +/- 0.010
	**BG10 *OCH1***	
**growth medium**	eGFP	DsRed
YPD	0.221 +/- 0-030	0.233 +/- 0-016
YPDN	0.196 +/- 0.005	0.195 +/- 0.003
BMD	0.236 +/- 0.004	0.230 +/- 0.009
BMDN	0.204 +/- 0.003	0.222 +/- 0.009

Table S2. **Growth rates, μ [h^{-1}] of ancestral GFP-and DsRed-expressing strains in all growth media used for subsequent experimental evolution.** Values represent averages +/- standard error of *n* = 4.

	fitness [ω] of GFP strain			
	**YPD**	**YPDN**	**BMD**	**BMDN**
**X-33**	1.07 +/- 0.07	1.15 +/- 0.02	1.09 +/- 0.13	1.11 +/- 0.02
**BG10**	1.18 +/- 0.07	1.16 +/- 0.07	1.34 +/- 0.05	1.30 +/- 0.09
**BG10 Δ*OCH1***	1.04 +/- 0.01	0.99 +/- 0.02	0.88 +/- 0.01	0.95 +/- 0.00

Table S3. **Fitness [ω] of ancestral GFP-expressing strains**. Fitness is in comparison with the ancestral DsRED-expressing *P. pastoris* strains used in the current study. Values represent average +/- standard deviation of *n* = 4.

	competition	
**medium**	**BG10 eGFP vs. ∆*OCH1* DsRed**	**BG10 DsRed / ∆*OCH1* eGFP**
**YPD**	0.956 +/- 0.019	2.899 +/- 0.130
**YPDN**	0.868 +/- 0.030	0.854 +/- 0.059
**BMD**	1.212 +/- 0.014	1.007 +/- 0.090
**BMDN**	1.189 +/- 0.067	0.770 +/- 0.029

Table S4 Table. **Fitness [ω] of ancestral BG10 strains as compared to ancestral BG10 ∆*OCH1* strains.** Values represent the mean of *n* = 3 competitions +/- standard deviation.

Growth medium	Population	X-33	BG10	BG10 ∆*OCH1*
**YPD**	Population 1	10.45	10.45	10.38
	Population 2	10.45	10.46	10.34
	Population 3	10.45	10.44	10.38
	Population 4	10.45	10.45	10.36
**YPDN**	Population 1	10.39	10.42	10.27
	Population 2	10.37	10.42	10.22
	Population 3	10.37	10.42	10.25
	Population 4	10.37	10.37	10.23
**BMD**	Population 1	10.35	10.46	10.40
	Population 2	10.41	10.41	10.39
	Population 3	10.42	10.40	10.39
	Population 4	10.35	10.42	10.42
**BMDN**	Population 1	10.28	10.42	10.32
	Population 2	10.32	10.40	10.33
	Population 3	10.29	10.43	10.32
	Population 4	10.29	10.39	10.34

Table S5. **Estimated number of CCD (10^x) for each of the evolved *P. pastoris* population.** CCD was calculated as described in in the Material and Methods sections of the main manuscript.

growth medium					
	**populations**	**YPD**	**YPDN**	**BMD**	**BMDN**
**X-33**	**YPD500**	126.1 +/- 12.6	129.5 +/- 24.3	87.4 +/- 1.8	100.5 +/- 4.6
	**YPDN500**	112.1 +/- 8.1	108.6 +/- 12.9	105.6 +/- 5.5	116.6 +/- 8.6
	**BMD500**	87.2 +/- 12.6	74.3 +/- 15.5	133.7 +/- 27.3	122.7 +/- 18.2
	**BMDN500**	104.5 +/- 16.8	74.3+/- 15.5	119.8. +/- 6.5	136.1 +/- 8.9
**BG10**	**YPD500**	109.1 +/- 8.3	164.1 +/- 52.0	118.2 +/- 7.1	106.3 +/- 4.5
	**YPDN500**	108.4 +/- 5.3	135.7 +/- 6.1	118.7 +/- 11.4	111.0 +/- 25.5
	**BMD500**	102.2 +/- 4.1	112.4 +/- 26.0	120.9 +/- 20.5	142.8 +/- 9.2
	**BMDN500**	116.1 +/- 15.0	94.4 +/- 59.3	115.4 +/- 3.5	133.7 +/-.9.9
**BG10 Δ*OCH1***	**YPD500**	101.6 +/- 10.3	101.0 +/- 17.8	99.1 +/- 7.0	95.1 +/- 2.9
	**YPDN500**	115.9 +/- 13.6	110.3 +/- 5.5	99.5 +/- 14.0	99.4 +/- 10.2
	**BMD500**	96.0 +/- 32.6	96.8 +/- 20.2	96.0 +/- 13.7	101.3 +/- 9.5
	**BMDN500**	74.9 +/- 20.8	106.2 +/- 21.9	107.5 +/- 15.0	105.8 +/- 11.4

Table S6. Maximum growth rates during exponential phase [% $\mu_{ancestor}$] of evolved *P. pastoris* populations in adaptive and non-adaptive conditions. Average growth rates +/- standard deviation from all 4 replicate populations measured in duplicates (*n* = 8).

growth medium					
	**populations**	**YPD**	**YPDN**	**BMD**	**BMDN**
**X-33**	**YPD500**	117.4 +/- 13.3	94.1 +/- 33.4	106.2 +/- 6.2	118.4 +/- 13.7
	**YPDN500**	87.6 +/- 11.1	129.5 +/- 27.5	88.7 +/- 36.5	141.3 +/- 12.8
	**BMD500**	104.4 +/- 5.8	142.1 +/- 12.3	72.2 +/- 41.2	61.9 /- 38.9
	**BMDN500**	120.4 +/- 4.7	148.5 +/- 11.7	107.8 +/- 29.9	67.0 +/- 32.4
**BG10**	**YPD500**	106.7 +/- 5.4	88.2 +/- 13.9	103.0 +/- 6.6	167.0 +/- 9.9
	**YPDN500**	94.6 +/- 12.5	235.4 +/- 13.6	91.3 +/- 20.8	62.4 +/- 23.4
	**BMD500**	86.7 +/- 9.2	80.5 +/- 15.6	131.7 +/- 11.9	150.3 +/- 31.3
	**BMDN500**	96.0 +/- 20.1	108.4 +/- 24.2	92.8 +/- 10.3	124.9 +/- 4.2
**BG10 Δ*OCH1***	**YPD500**	121.9 +/- 8.7	73.1 +/- 10.8	75.2 +/- 19.2	43.6 +/- 36.1
	**YPDN500**	71.4 +/- 13.1	76.7 +/- 7.8	132 .0+/- 31.0	78.5 +/- 23.7
	**BMD500**	109.7 +/- 14.7	90.4 +/- 12.0	66.7 +/- 28.9	70.5 +/- 19.7
	**BMDN500**	120.0 +/- 12.1	66.0 +/- 17.9	70.9 +/- 11.5	122.9 +/-14.2

Table S7. Early growth rates [% $\mu_{ancestor}$] from t = 0 to 2 hours after inoculation of evolved *P. pastoris* populations in adaptive and non-adaptive conditions. Average growth rates +/- standard deviation from all 4 replicate populations measured in duplicates (*n* = 8).

strain	clone	coverage	mapped	% mapped	properly paired	% properly paired	singletons	% singletons	% genome covered
X-33	**YPD GFP 1a**	71	2664663	81.43%	2559838	78.64%	60974	1.87%	91,11
	**YPD RED 1b**	75	2820130	82.41%	2710842	79.78%	53594	1.58%	91,12
	**YPD GFP 2b**	72	2771943	85.00%	2611892	81.19%	53112	1.65%	91,09
	**YPD RED 2b**	81	3064606	84.26%	2938400	81.43%	59031	1.64%	91,11
	**YPDN GFP 1c**	77	2893073	85.01%	2783058	82.34%	55823	1.65%	91,12
	**YPDN RED 1c**	66	2510193	78.94%	2420606	76.48%	56242	1.78%	91,09
	**YPDN GFP 2b**	68	2543747	78.09%	2446180	75.45%	64889	2.00%	91,14
	**YPDN RED 2b**	98	3660172	79.11%	3529132	76.58%	90618	1.97%	91,16
	**BMD GFP 1a**	81	3030632	79.67%	2922324	77.18%	64395	1.70%	91,1
	**BMD RED 1b**	67	2553851	78.53%	2464322	76.12%	58431	1.80%	91,16
	**BMD GFP 2b**	81	3084847	79.09%	2998618	77.20%	52575	1.35%	91,11
	**BMD RED 2c**	54	2063370	78.31%	1978066	75.54%	51377	1.96%	91,09
	**BMDN GFP 1a**	86	3273737	73.43%	3148668	70.90%	87233	1.96%	91,09
	**BMDN RED 1a**	70	2631844	78.66%	2540866	76.30%	56574	1.70%	91,12
	**BMDN GFP 2a**	48	1816762	79.76%	1748922	77.10%	45865	2.02%	91,12
	**BMDN RED 2c**	69	2585886	81.57%	2510772	79.50%	49647	1.57%	91,13
BG10	**YPD GFP1c**	53	2001716	91.93%	1966272	90.76%	16176	0.75%	91,12
	**YPD RED1a**	60	2254029	90.80%	2219370	89.70%	18251	0.74%	91,1
	**YPD GFP2c**	59	2237515	91.11%	2204714	90.10%	16300	0.67%	91,1
	**YPD RED2c**	61	2299644	90.71%	2264960	89.64%	17976	0.71%	91,13
	**YPDN GFP1b**	56	2132540	94.01%	2092680	92.66%	18287	0.81%	91,14
	**YPDN RED1b**	61	2334947	90.88%	2281938	89.18%	27812	1.09%	91,12
	**YPDN GFP2c**	33	1291422	91.58%	1265734	90.11%	14114	1.00%	91,09
	**YPDN RED2c**	51	1965366	90.85%	1919702	89.08%	26562	1.23%	91,1
	**BMD GFP1c**	49	1847041	89.99%	1813780	88.74%	16363	0.80%	91,09
	**BMD RED1c**	54	2078412	87.54%	2032974	85.98%	25341	1.07%	91,16
	**BMD GFP2a**	48	1834157	89.81%	1805502	88.72%	15020	0.74%	91,14
	**BMD RED2c**	47	1792500	85.54%	1750036	83.84%	26212	1.26%	91,13
	**BMDN GFP1b**	47	1775367	90.14%	1746170	89.03%	14386	0.73%	91,12
	**BMDN RED1c**	46	1788519	85.47%	1746294	83.78%	24506	1.18%	91,12
	**BMDN GFP 2c**	40	1511523	89.03%	1486908	87.91%	12534	0.74%	77,6
	**BMDN RED2b**	47	1782214	88.48%	1749532	87.21%	16402	0.82%	91,11
BG10	**YPD RED 1c**	48	1872063	87.64%	1821760	85.61%	30428	1.43%	91,07
Δ*OCH1*	**YPD GFP 1a**	42	1606320	89.43%	1577670	88.19%	15245	0.85%	91,1
	**YPD RED 2c**	58	2207075	89.04%	2156316	87.34%	26498	1.07%	91,08
	**YPD GFP 2c**	47	1793946	88.78%	1763648	87.63%	15852	0.79%	91,09
	**YPDN RED 1a**	56	2120333	89.65%	2063510	87.63%	30837	1.31%	91,1
	**YPDN GFP 1a**	55	2079675	89.97%	2037092	88.55%	20463	0.89%	91,12
	**YPDN RED 2b**	38	1439110	89.10%	1413830	87.85%	13409	0.83%	91,08
	**YPDN GFP 2b**	51	1941082	86.75%	1899986	85.28%	23164	1.04%	91,08
	**BMD RED 1c**	28	1056749	89.62%	1029048	87.58%	17159	1.46%	91,09
	**BMD GFP 1b**	55	2107001	89.30%	2057074	87.50%	29087	1.24%	91,1
	**BMD RED 2b**	61	2352281	86.55%	2306326	85.14%	27092	1.00%	91,11
	**BMD GFP 2b**	55	2085305	91.21%	2046372	89.87%	18860	0.83%	91,11
	**BMDN RED 1a**	49	1848613	92.12%	1823310	91.17%	12694	0.63%	91,09
	**BMDN GFP 1c**	52	1969505	92.31%	1941602	91.33%	13691	0.64%	91,09
	**BMDN RED 2a**	53	2014884	91.72%	1988122	90.82%	13081	0.60%	91,1
	**BMDN GFP 2b**	53	2007531	92.32%	1977918	91.30%	14321	0.66%	91,1

Table S8. Illumina MiSeq statistics of the 48 evolved *P. pastoris* genomes.

clone	chr	pos	type	ref	alt	gene/locus	effect
**YPD GFP1a**	chr. 1	1910190	Indel	CT	C	PAS_chr1-4_0279 (SSK2)	stop at amino acid position 1086
**YPD RED1b**	chr. 1	1910860	Indel	GC	G	PAS_chr1-4_0279 (SSK2)	stop at amino acid position 862
**YPD GFP2b**	chr. 1	747966	SNP	G	A	HOG4/SSK4 MAP Kinase	stop codon
**YPD RED2b**	chr. 1	1909967	Indel	GT	G	PAS_chr1-4_0279 (SSK2)	stop at amino acid position 1160
	chr. 3	843411	SNP	G	C	TPS1 related / YDR072C	W233 to S233
	chr. 3	1390423	Indel	C	CTGTTGT	FR839630.1 / XM_002492621 / PUF4-like	pos 571 2 Q insertion (Q repeat region) pumilio-like repeat?
**YPDN GFP1c**	chr. 4	1096585	SNP	G	A	FR839631.1 / Pho84	G159 to D159
**YPDN RED1c**	chr.2	2261762	SNP	C	T	PAS_chr2-1_0073 (RIM21)	S287 to F287
**YPDN GFP2b**	chr. 1	1350300	SNP	T	C	PAS_chr1-1_0428 / ENA2 / P-type ATPase Sodium pump	N93 to D93
	chr. 2	1951595	SNP	C	G	PAS_chr2-1_0235 (Pho89)	G134 to R134
	chr. 4	1097181	SNP	G	A	FR839631.1 / Pho84	G358 to R 358
**YPDN RED2b**	chr. 1	1347944	SNP	G	A	PAS_chr1-1_0428 (ENA2)	P878 to L878
	chr. 2	623956	SNP	G	T	PAS_chr2-2_0324 (GYL1)	A39 to S39
	chr. 3	1058830	Indel	A	AACTATCT	PAS_chr3_0625 (RIM101)	frameshift and premature stop
	chr. 4	1096215	SNP	C	T	FR839631.1 / Pho84	Q36 to stop
**BMD GFP1a**	chr. 3	962181	Indel	GC	G	PAS_chr3_0669 (fungal MHR superfamily)	changed c-terminus and premature stop after 100aa
**BMD RED1b**	chr. 1	1703984	SNP	G	A	PAS_chr1-4_0160 (HIS4)	G557 to S557
	chr. 3	313639	SNP	A	G	PAS_chr3_1001 (TUP1)	H467 to R467
**BMD GFP2b**	chr. 1	1703984	SNP	G	A	PAS_chr1-4_0160 (HIS4)	G557 to S557
	chr. 1	2682926	SNP	G	A	PAS_FragB_0008 (PDE2)	W458 to stop
	chr. 2	174635	SNP	C	T	XP_002492122.1 / MRPS5 and XP_002492121.1 / ARF1	non-coding upstream of mitochondrial ribosomal subunit and ARF1 GTPase
**BMD RED2c**	chr. 1	1704727	SNP	C	A	PAS_chr1-4_0160 (HIS4)	D804 to E804
	chr. 2	1993177	Indel	GT	G	PAS_chr2-1_0214 cytc reductase complex	changed c-terminus and premature stop after 1042aa
	chr. 3	2124206	SNP	C	T	PAS_chr3_0052	G175 to D175 thiamine pyrophosphokinase
**BMDN GFP1a**	chr. 3	960331	SNP	C	A	PAS_chr3_0669 (fungal MHR superfamily)	S700 to I700
	chr. 3	1331718	Indel	AT	A	intergenic PAS_chr3_0480 (XDJ1) and PAS_chr3_0478 (RRN11)	downstream of XDJ1 and upstream of RRN11

	chr. 3	2115746	Indel	GA	G	PAS_chr3_0061 / TUL1	frame shift stop codon at position 74
**BMDN RED1a**	chr. 3	312675	SNP	C	T	PAS_chr3_1001 (TUP1)	Q146 to stop codon
	chr. 3	1178447	SNP	C	T	PAS_chr3_0560(MSB1)	T1146 to I1146 at protein c-terminus
	chr. 3	1316342	SNP	G	A	PAS_chr3_0488	G729 synonymous mutation
	chr. 3	1743772	SNP	G	A	PAS_chr3_0247 (CAC2)	P345 synonymous mutation
**BMDN GFP2a**	chr. 1	2036266	Indel	AT	A	PAS_chr1-4_0672 (CYB5)	frameshift stop at pos. 21
	chr. 3	959553	SNP	C	T	PAS_chr3_0669 (fungal MHR superfamily)	premature stop at position 959
**BMDN RED2c**	chr. 1	2455746	SNP	A	G	intergenic PAS_chr1-4_0565 (CUP2) PAS_chr1-4_0567 (PRE4)	downstream of both open reading frames
	chr. 2	1845389	SNP	A	G	PAS_chr2-1_0297 (SEC15)	M304 to V304
	chr. 3	313098	SNP	G	T	PAS_chr3_1001 (TUP1)	V287 to F287
	chr. 3	1469802	Indel	AC	A	intergenic g2867 (unknown hypothetical) and PAS_chr3_0397 (CDC11)	noncoding between g2867 (downstream) and g2868 (upstream)
	chr. 4	526143	SNP	G	T	PAS_chr4_0649 (UBX3-like)	stop at position 271

Table S9. **Mutations identified in the evolved *P. pastoris* X-33 clones**. For each mutation the chromosomal location (chr) and nucleotide position (pos) is shown with respect to the CBS 7435 reference genome. The type of mutation (Indel / SNP), reference sequence (ref) and sequence in the evolved clone (alt), as well as the affected locus (gene/locus) and effect are shown. Recurrent mutational targets are highlighted by different colors.

clone	chr	pos	type	ref	alt	gene/locus	effect
**YPD GFP1c**	chr. 1	2375201	SNP	G	T	PAS_chr1-4_0530 (SOK2)	R339 to S339
	chr. 2	219784	Indel	TG	T	intergenic PAS_chr2-2_0111 (GPD1)	upstream of GPD1
**YPD RED1a**	chr. 1	1910627	Indel	GA	G	PAS_chr1-4_0279 (SSK2)	frameshift stop at position 940
	chr. 4	1121333	SNP	T	A	PAS_chr4_0327 (GCN1)	L2712 to I2712
**YPD GFP2c**	chr. 1	1911735	SNP	C	A	PAS_chr1-4_0279 (SSK2)	stop at position 562
**YPD RED2c**	chr. 1	2056661	SNP	C	T	upstream of PAS_chr1-4_0349 (PEX8) and PAS_chr1-4_0350 (DSC2)	-
	chr. 2	2100084	SNP	T	A	PAS_chr2-1_0162 (SLN1)	N495 to I495
**YPDN GFP1b**	chr. 1	818136	SNP	T	G	PAS_chr1-1_0139 (SPC7)	L570 to V570
	chr. 2	485383	SNP	C	T	PAS_chr2-2_0258 (EMP70 domain)	P638 to L638
	chr. 2	2261762	SNP	C	T	PAS_chr2-1_0073 (RIM21)	S287 to F287
	chr. 4	1097664	Indel	GC	G	PAS_chr4_0337 (PHO84)	frameshift stop at position 524
**YPDN RED1b**	chr. 1	359814	Indel	T	TGG	upstream of PAS_chr1-3_0192 (RAM2)	-
	chr. 2	848176	SNP	C	T	integenic	-
	chr. 4	1096513	Indel	GC	G	PAS_chr4_0337 (PHO84)	stop at position 135
	chr. 4	1410744	SNP	C	T	PAS_chr4_0184 (RIM8)	S80 to L80
**YPDN GFP2c**	chr. 2	219326	SNP	A	T	PAS_chr2-2_0111	L104 to Q104
	chr. 2	2261090	SNP	C	T	PAS_chr2-1_0073 (RIM21)	P63 to L63
	chr. 3	1577636	SNP	G	A	PAS_chr3_0340 (eIF3)	R45 to K45
	chr. 4	1096510	Indel	AGT	A	PAS_chr4_0337 (PHO84)	frameshift stop at position 138
**YPDN RED2c**	chr. 3	1058688	Indel	A	AATTCCAACTC	PAS_chr3_0625 (RIM101)	frameshift stop at position 513
**BMD GFP1c**	chr. 2	1760403	SNP	C	G	PAS_chr2-1_0339, similar to *S. cerevisiae* YLR177W	S20 to stop
**BMD RED1c**	chr. 1	2682901	Indel	CG	C	PAS_FragB_0008 (PDE2)	frameshift stopp at position 469
	chr. 3	960756	Indel	GC	G	PAS_chr3_0669 MHR-like	-
**BMD GFP2a**	chr. 1	2682538	Indel	T	TTAAA	PAS_FragB_0008 (PDE2)	frameshift stop at position 343
	chr. 3	961799	Indel	GT	G	PAS_chr3_0669 MHR-like	frameshift stop at position 224
**BMD RED2c**	chr. 1	1910249	SNP	A	G	PAS_chr1-4_0279 (SSK2)	L1057 to S1057
**BMDN GFP1b**	chr. 3	312675	SNP	C	T	PAS_chr3_1001 (TUP1 transcriptional repressor)	stop at position 146
**BMDN RED1c**	chr. 4	526418	SNP	C	A	PAS_chr4_0649 (UBX3-like)	stop at position 180
**BMDN GFP2c**	chr. 1	2875345	Indel	-	-	5582 bp deletion unknown hypothetical orf	-
	chr. 3	312687	SNP	C	T	PAS_chr3_1001 (TUP1 transcriptional repressor)	stop at position 150
**BMDN RED2b**	chr. 3	2114593	Indel	G	GA	PAS_chr3_0061 (TUL1)	frameshift stop at position 472

Table S10. **Mutations identified in the evolved *P. pastoris* BG10 clones.** For each mutation the chromosomal location (chr) and nucleotide position (pos) is shown with respect to the CBS 7435 reference genome. The type of mutation (Indel / SNP), reference sequence (ref) and sequence in the evolved clone (alt), as well as the affected locus (gene/locus) and effect are shown. Recurrent mutational targets are highlighted by different colors.

clone	contig	pos	type	ref	alt	gene/locus	effect
**YPD RED1c**	chr. 2	936257	indel	C	CA	upstream of PAS_chr2-1_0765 (FLC2) and XM_002491656.1 (ACS1)	downregulation FLC2
**YPD GFP1a**	chr. 2	937030	SNP	G	A	upstream of PAS_chr2-1_0765 (FLC2) and XM_002491656.1 (ACS1)	downregulation FLC2
	chr. 3	600924	indel	GT	G	SSK1	SSK1 frameshift and stop at aa53
**YPD RED2c**	chr. 2	936810	indel	AC	A	upstream of PAS_chr2-1_0765 (FLC2) and XM_002491656.1 (ACS1)	downregulation FLC2
**YPD GFP2c**	chr. 1	206601	indel	C	CCAAACTA	PAS_chr1-3_0106 (NPR1)	frameshift stop at aa300
	chr. 2	936817	indel	GT	G	upstream of PAS_chr2-1_0765 (FLC2) and XM_002491656.1 (ACS1)	downregulation FLC2
	chr. 2	1372143	SNP	T	C	upstream of PAS_chr2-1_0551 (EMC3) downstream of PAS_chr2-1_0552 (ADD66)	-
	chr. 3	600283	indel	GC	G	SSK1	SSK1 frameshift and stop at aa241
**YPDN RED1a**	chr. 3	1058511	SNP	C	T	PAS_chr3_0625 (RIM101)	Q440 to stop
**YPDN GFP1a**	chr. 1	2779984	SNP	C	A	PAS_FragB_0063 (PFS2)	D187 to E187
	chr. 3	600924	indel	GT	G	SSK1	SSK1 frameshift and stop at aa53
**YNPD RED2b**	chr. 2	2261762	SNP	C	T	PAS_chr2-1_0073 (RIM21)	S287 to F287
	chr. 3	600794	indel	AT	A	SSK1	SSK1 frameshift and stop at aa53
**YPDN GFP2b**	chr. 1	1347883	SNP	C	T	PAS_chr1-1_0428 / ENA2 / P-type ATPase Sodium pump	M898 to I898
**BMD RED1c**	chr. 3	1918359	indel	TA	T	IRA1	frameshift stop at aa953
	chr. 4	1495	SNP	A	C	PAS_chr4_0879 (SLA2)	
**BMD GFP1b**	chr. 3	1920040	indel	TA	T	IRA1	frameshift stop at aa1504
**BMD RED2b**	chr. 3	600794	indel	AT	A	SSK1	SSK1 frameshift and stop at aa53
	chr. 3	1918078	indel	TA	T	IRA1	frameshift stop at aa849
**BMD GFP2b**	chr. 3	1918621	indel	GT	G	IRA1	frameshift stop at aa1037
**BMDN RED1a**	chr. 1	1910362	SNP	A	T	PAS_chr1-4_0279 (SSK2)	C1019 to stop
	chr. 2	474950	SNP	A	T	upstream of PAS_chr2-2_0482 (FLO11)	
**BMDN GFP1c**	chr. 1	1910843	Indel	GA	G	PAS_chr1-4_0279 (SSK2)	frameshift stop at aa865
**BMDN RED2a**	chr. 1	1910362	SNP	A	T	PAS_chr1-4_0279 (SSK2)	C1019 to stop
**BMDN GFP2b**	chr. 1	1909882	SNP	A	C	PAS_chr1-4_0279 (SSK2)	Y1179 to stop

Table S11. Mutations identified in the evolved *P. pastoris* BG10 Δ*OCH1* clones. For each mutation the chromosomal location (chr) and nucleotide position (pos) is shown with respect to the CBS 7435 reference genome. The type of mutation (Indel / SNP), reference sequence (ref) and sequence in the evolved clone (alt), as well as the affected locus (gene/locus) and effect are shown. Recurrent mutational targets are highlighted by different colors.

Mutation	type		No.	%
**Indels**	insertions		6	**17.1**
	deletions		29	**82.9**
**SNPs**	G	A	11	**19.6**
	G	T	4	**7.1**
	G	C	1	**1.8**
	C	A	5	**8.9**
	C	T	18	**32.1**
	C	G	2	**3.6**
	A	G	4	**7.1**
	A	C	2	**3.6**
	A	T	4	**7.1**
	T	G	1	**1.8**
	T	C	2	**3.6**
	T	A	2	**3.6**

Table S12. **Mutation statistics.** Total number of Insertion-Deletions (Indels) and single nucleotide polymorphisms (SNPs) identified in the 48 sequenced *P. pastoris* clones.

clone	YPD	YPDN	BMD	BMDN
**X33 YPD GFP1a***	109.6 +/- 1.5	65.2 +/- 4.9	98.2 +/- 1.3	100.8 +/- 2.2
**X33 YPD GFP2b**[†]	111.1 +/- 1.1	39.7 +/- 8.9	97.7 +/- 4.5	102.1 +/- 0.3
**X33 BMD GFP1a**[a]	107.8 +/- 2.0	71.7 +/- 2.9	115.6 +/- 3.8	120.5 +/- 2.1
∆***OCH1* YPD RED1c**[b]	101.7 +/- 4.3	66.3 +/- 5.4	96.0 +/- 10.6	84.1 +/- 4.9
∆***OCH1* YPD RED2c**[b]	111.8 +/- 1.8	67.7 +/- 14.8	98.7 +/- 5.1	71.5 +/- 4.1
∆***OCH1* YPD GFP1a**[c]	170.8 +/- 3.4	125.2 +/- 10.6	118.4 +/- 1.6	117.9 +/- 1.7
∆***OCH1* YPD GFP2c**[c]	155.7 +/- 0.8	124.0 +/- 10.0	95.2 +/- 3.6	109.2 +/- 2.1
∆***OCH1* BMD GFP2b**[d]	103.5 +/- 16.5	142.5 +/- 10.2	117.1 +- 8.4	110.0 +/- 4.3
∆***OCH1* BMDN GFP1c**[e]	149.9 +/- 2.2	122.9 +/- 8.4	104.6 +/- 4.9	117.3 +/- 2.6

Table S13. **Relative growth rate [% $\mu_{ancestor}$] of selected evolved single clones of *P. pastoris* X-33 and BG10 ∆*OCH1* in all 4 growth environments.** * single SSK2 mutation [†] single *SSK4* mutation [a] single PAS-ch3_0669 mutation [b] single intergenic *ACS1*/*FLC2* mutations [c] *OCH1* deletion clones with an additional mutations; [d] single *IRA1* mutation; [e] single *SSK2* mutation; values represent the mean of *n* = 3 +/- standard deviation.

clone	YPD	YPDN	BMD	BMDN
**X33 YPD GFP1a***	93.8 +/- 0.6	50.3 +/- 0.4	87.1 +/- 1.4	84.9 +/- 4.6
**X33 YPD GFP2b†**	90.4 +/- 1.7	49.7 +/- 4.6	90.5 +/- 2.3	85.4 +/- 1.1
**X33 BMD GFP1a[a]**	102.6 +/- 9.8	82.9 +/- 5.2	81.5 +/- 4.7	90.6 +/- 4.1
**Δ*OCH1* YPD RED1c[b]**	90.6 +/- 4.2	94.0 +/- 0.4	95.0 +/- 1.2	98.0 +/-2.6
**Δ*OCH1* YPD RED2c[b]**	91.7 +/- 1.9	86.2 +/- 0.0	105.2 +/- 2.3	109.6 +/- 5.4
**Δ*OCH1* YPD GFP1a[c]**	124.9 +/- 5.5	78.1 +/- 5.3	86.7 +/- 2.0	89.9 +/- 5.9
**Δ*OCH1* YPD GFP2c[c]**	121.1 +/- 1.6	80.4 +/-4.9	105.3 +/- 4.7	100.3 +/-2.4
**Δ*OCH1* BMD GFP2b[d]**	89.1 +/- 5.7	88.7 +/- 5.3	83.1 +/- 1.7	85.1 +/- 2.5
**Δ*OCH1* BMDN GFP1c[e]**	115.2 +/-7.7	74.6 +/- 4.9	80.9 +/- 5.2	84.8 +/- 1.2

Table S14. **Maximum OD_{600} [% ancestor] of selected evolved single clones of *P. pastoris* X-33 and BG10 Δ*OCH1* in all 4 growth environments**; * single SSK2 mutation † single *SSK4* mutation [a] single PAS-ch3_0669 mutation [b] single intergenic *ACS1*/*FLC2* mutations [c] *OCH1* deletion clones with an additional mutations; [d] single *IRA1* mutation; [e] single *SSK2* mutation; values represent the mean of *n* = 3 +/- standard deviation.

clone	YPD	YPDN	BMD	BMDN
**X33 YPD GFP1a***	1.201 +/- 0.075	0.842 +/- 0.013	0.919 +/- 0.006	0.748 +/- 0.007
**X33 YPD GFP2b†**	1.373 +/- 0.115	0.627 +/- 0.095	0.833 +/- 0.011	0.837 +/- 0.049
**X33 BMD GFP1a[a]**	1.010 +/- 0.034	0.704 +/- 0.001	1.107 +/- 0.019	1.300 +/- 0.101
**Δ*OCH1* YPD RED1c[b]**	1.336 +/- 0.004	1.017 +/- 0.009	0.890 +/- 0.009	0.797 +/- 0.010
**Δ*OCH1* YPD RED2c[b]**	1.615 +/- 0.039	1.066 +/- 0.006	0.939 +/- 0.008	0.858 +/- 0.009
**Δ*OCH1* YPD GFP1a[c]**	1.038 +/- 0.058	1.203 +/- 0.040	1.466 +/- 0.035	1.388 +/- 0.021
**Δ*OCH1* YPD GFP2c[c]**	1.222 +/- 0.003	1.282 +/- 0.140	1.363 +/- 0.029	1.224 +/- 0.038
**Δ*OCH1* BMD GFP2b[d]**	0.333 +/- 0.031	1.193 +/- 0.031	1.647 +/- 0.030	1.632 +/- 0.007
**Δ*OCH1* BMDN GFP1c[e]**	0.938 +/- 0.043	1.149 +/- 0.031	1.310 +/- 0.034	1.389 +/- 0.003

Table S15. **Competitive fitness [ω] relative to the ancestral strains of selected evolved single clones.** *P. pastoris* X-33 and BG10 Δ*OCH1* clones as compared to ancestral strains in all 4 growth environments; * single SSK2 mutation † single *SSK4* mutation [a] single PAS-ch3_0669 mutation [b] single intergenic *ACS1*/*FLC2* mutations [c] *OCH1* deletion clones with an additional mutations; [d] single *IRA1* mutation; [e] single *SSK2* mutation; values represent the mean of *n* = 4 +/- standard error.

Supplemental Figures

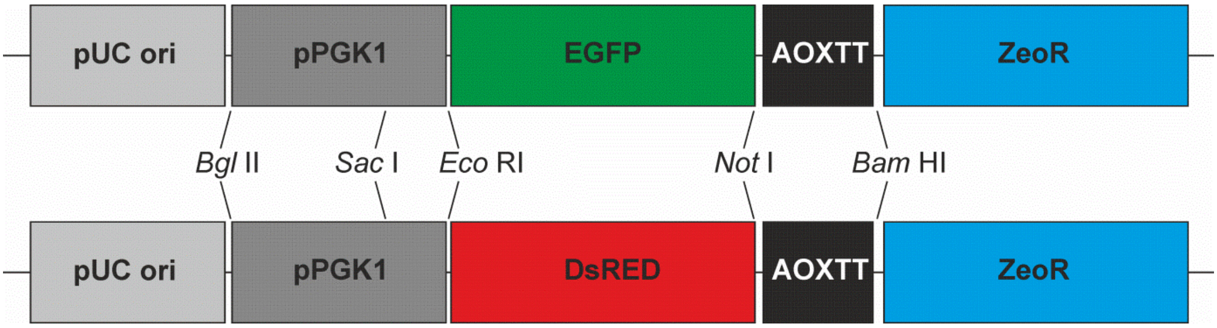

Figure S1. **Reporter gene constructs**. Vectors are based on a modified pGAPzB vector backbone, where the P_{GAP} has been replaced with P_{PGK1}. See also Table S1 for oligonucleotides used for plasmid construction.

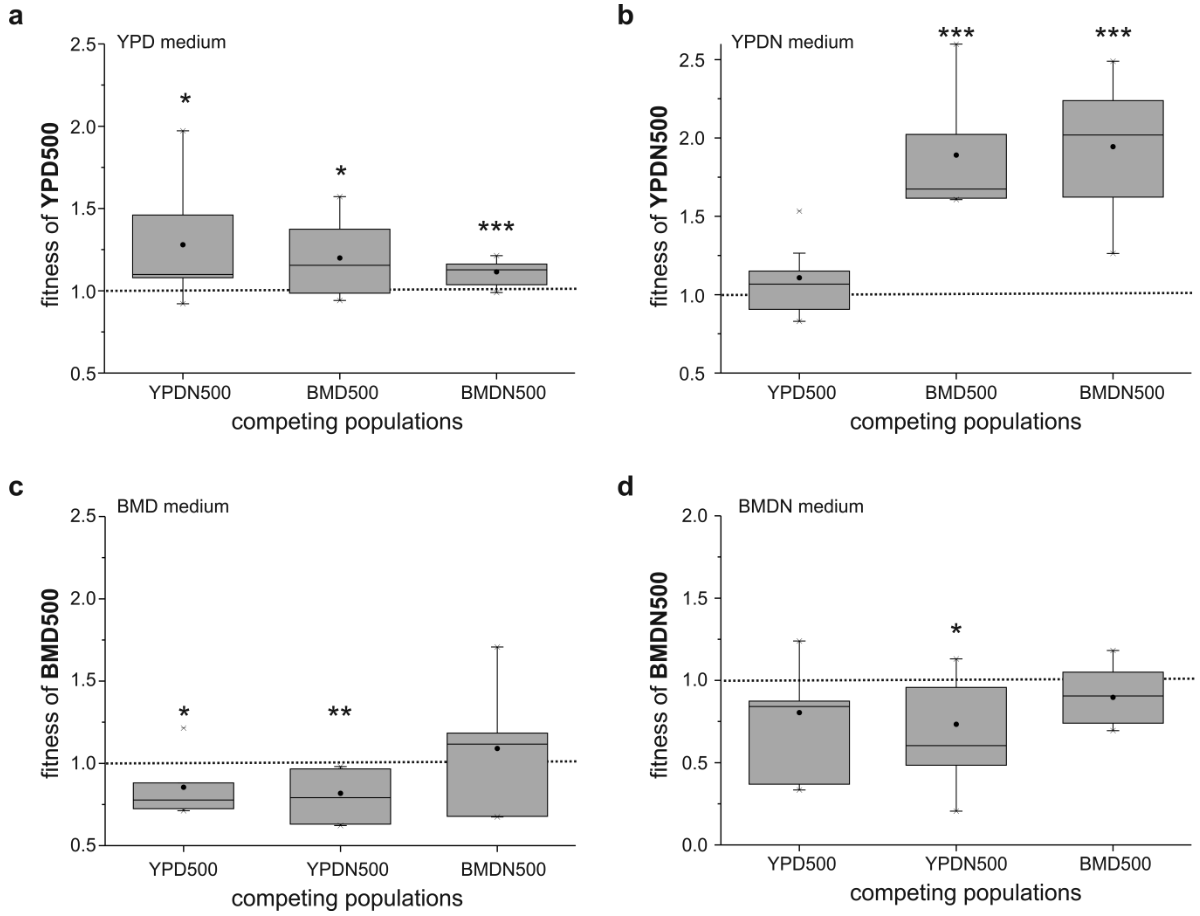

Figure S2. **Direct competition assays of evolved *P. pastoris* X-33 populations.** Fitness of YPD-evolved populations on YPD (a), YPDN-evolved on YPDN (b), BMD-evolved on BMD (c) and BMDN-evolved populations on BMDN (d) as compared with the remainder evolved populations. All possible pair-wise competitions were performed in duplicate (n = 8); paired Student's T-test values * $p \leq 0.05$, ** $p \leq 0.01$ and *** $p \leq 0.001$.

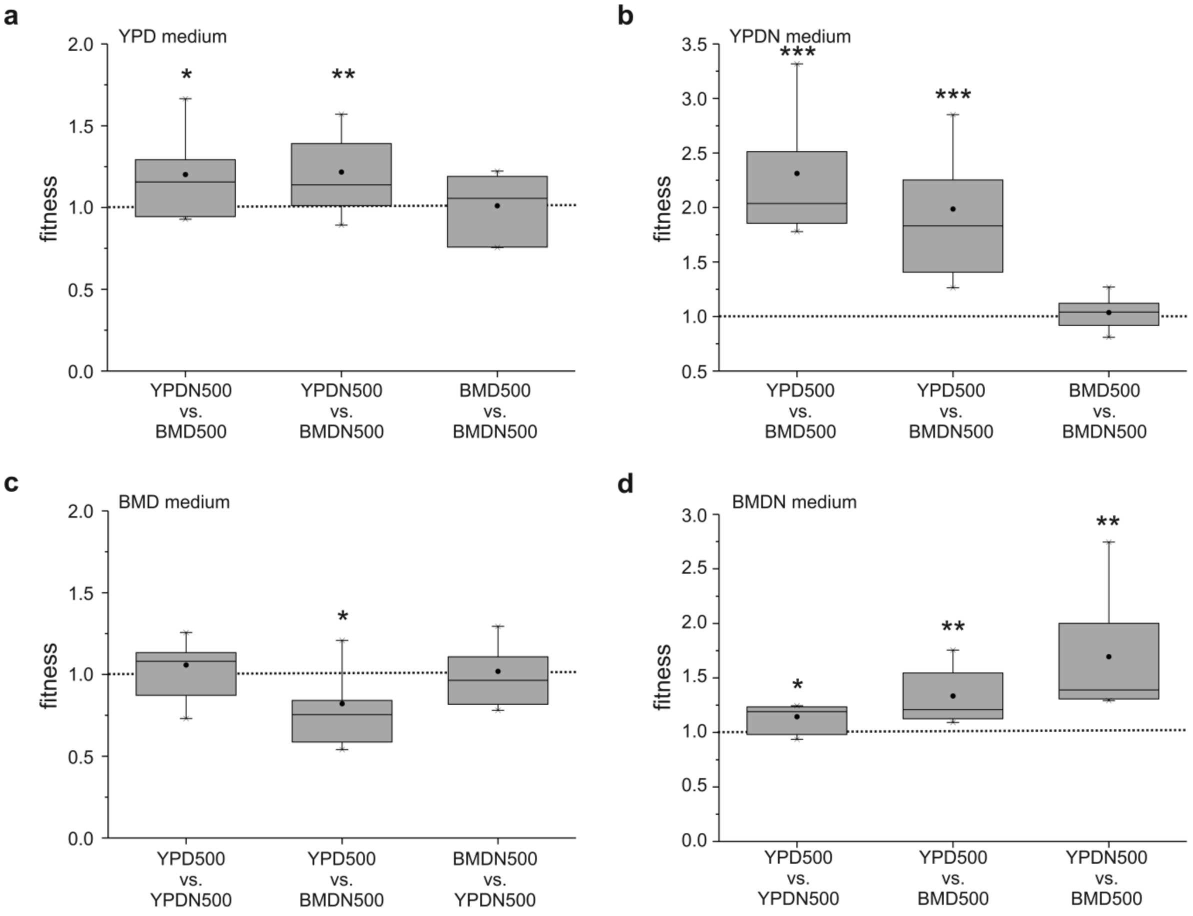

Figure S3. **Direct competition assays among evolved *P. pastoris* X-33 populations.** Competitions on (a) YPD, (b) YPDN, (c) BMD and (d) BMDN are shown. All possible pair-wise competitions were performed in duplicate (n = 8); paired Student's T-test values * $p \le 0.05$, ** $p \le 0.01$ and *** $p \le 0.001$.

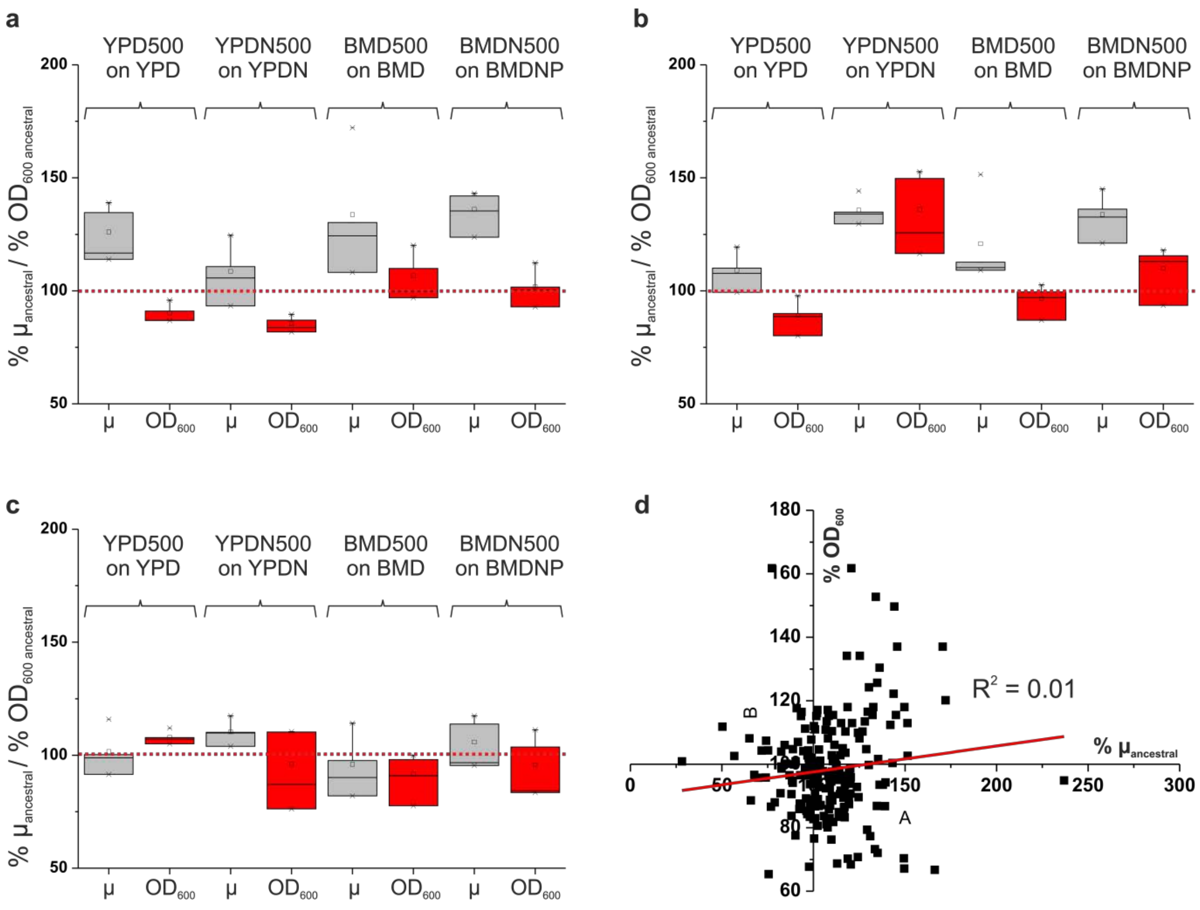

Figure S4. **Growth rates [% $\mu_{ancestral}$, grey boxplosts] and biomass yield [$\%_{ancestral}$ OD_{600}, red boxplots] of evolved *P. pastoris* populations.** (a) *P. pastoris* X-33; (b) BG10 and (c) BG10 Δ*OCH1* (d) overall correlation of growth rate and biomass yield (final OD_{600}) of growth tests performed in this study (n = 220).

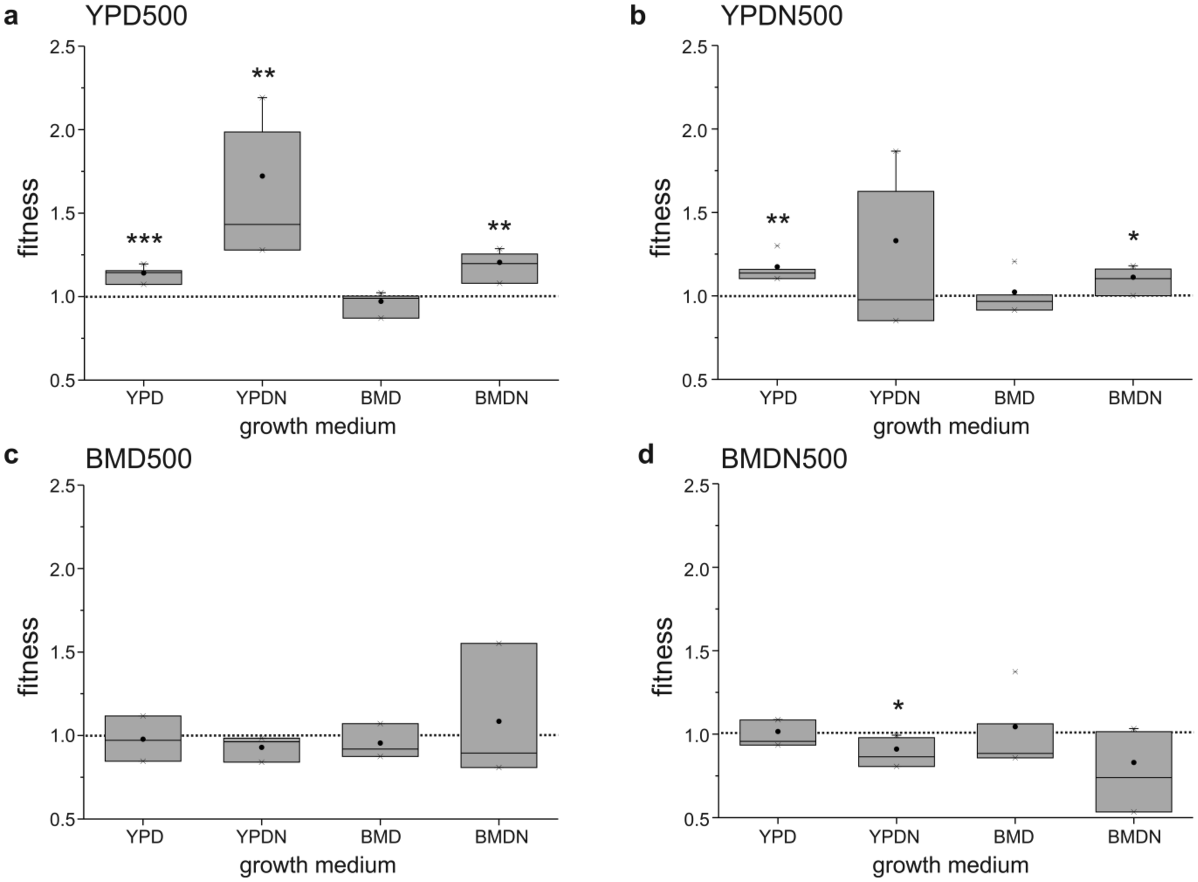

Figure S5. **Competitive fitness [ω] of evolved *P. pastoris* X-33 populations in different growth environments as compared with ancestral strains.** Fitness of YPD-evolved (a), YPDN-evolved (b), BMD-evolved (c) and BMDN-evolved populations (d) is shown. All possible pair-wise competitions were performed in duplicate (n = 8); paired Student's T-test values * $p \leq 0.05$, ** $p \leq 0.01$ and *** $p \leq 0.001$.

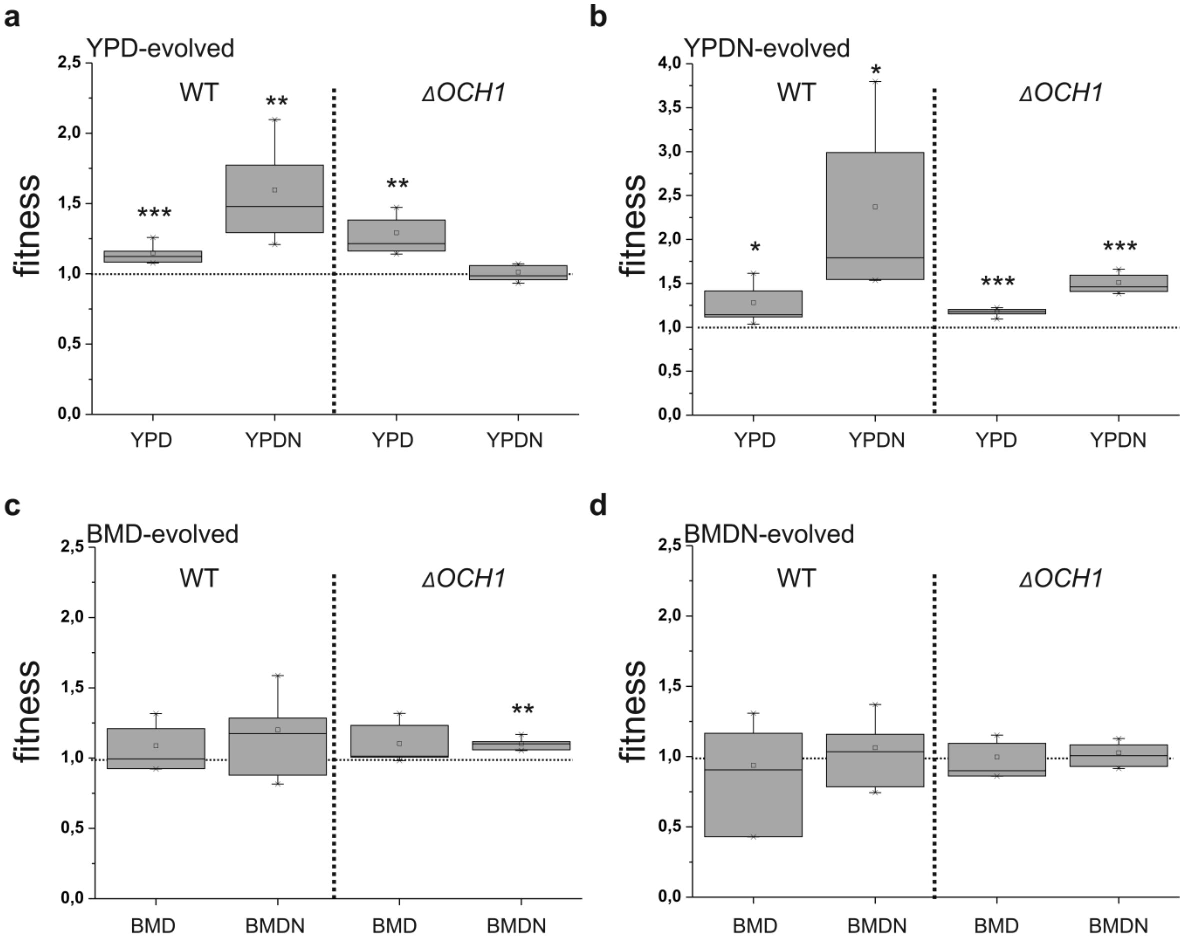

Figure S6. **Competitive fitness [ω] of evolved *P. pastoris* BG10 and BG10 Δ*OCH1* populations in different growth environments as compared with the ancestral strains.** YPD-evolved (a), YPDN-evolved (b), BMD-evolved (c) and BMDN-evolved (d); All possible pair-wise competitions were performed in duplicate (n = 8); paired Student's T-test values * $p \leq 0.05$, ** $p \leq 0.01$ and *** $p \leq 0.001$.

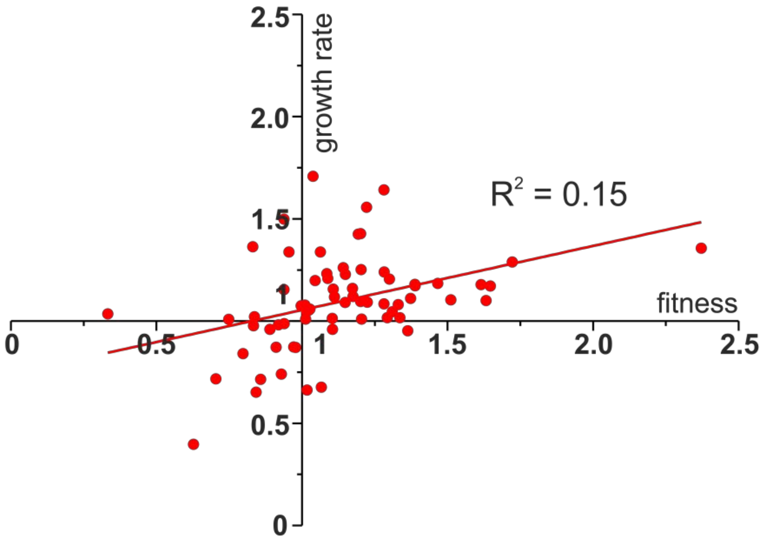

Figure S7. **Correlation of maximum growth rate and competitive fitness.** Correlation plot of competitive fitness and relative ancestral growth rates of all tests performed in the current study.

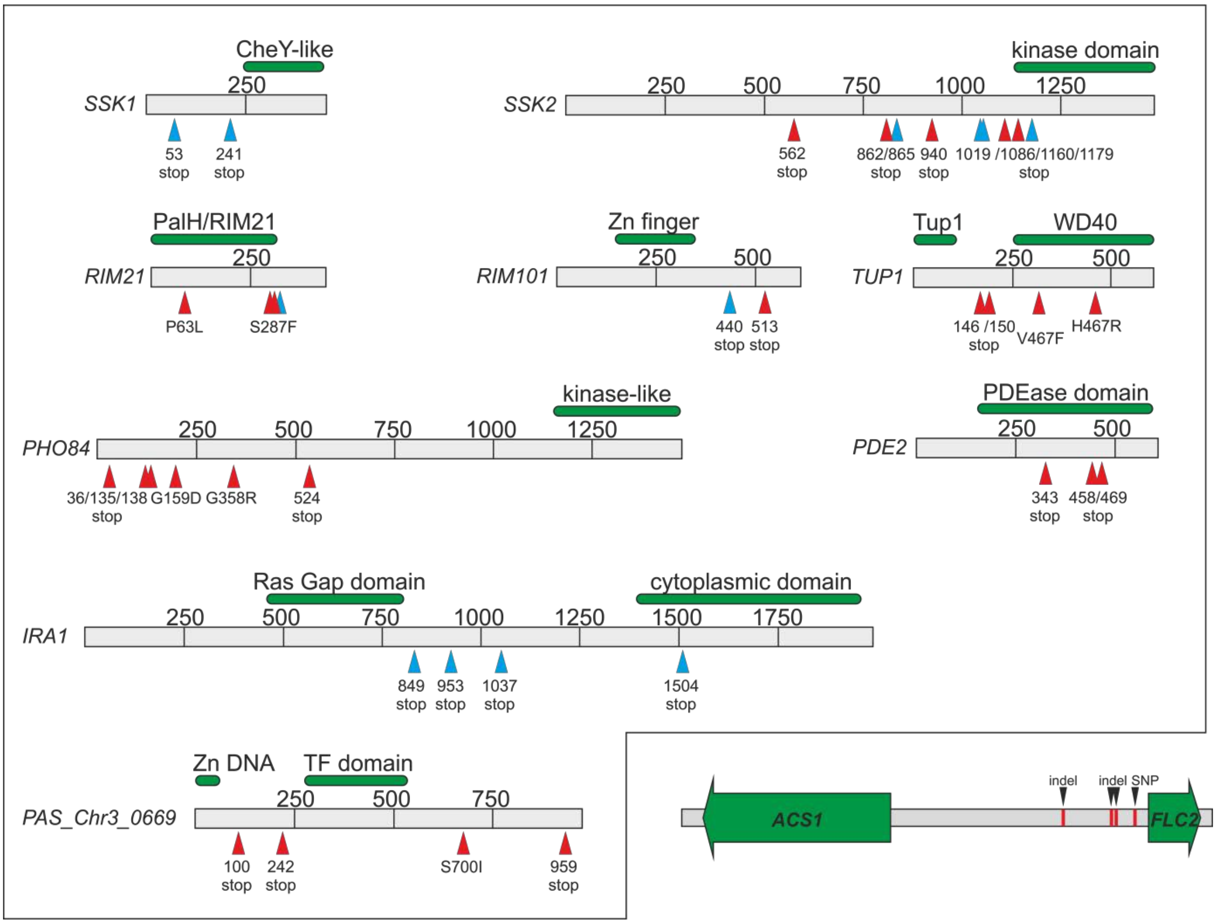

Figure S8. Selected recurrent mutational targets isolated from *P. pastoris* populations after 500 generations of laboratory evolution. Linear representations of proteins encoded by the corresponding ORFs are shown. Important protein domains as well as positions and types of mutation are indicated by arrows. Red – mutations in X-33 and BG10 genetic background and blue – mutations identified in evolved BG10 Δ*OCH1* clones. For the non-coding intergenic recurrent mutations in the *ACS1/FLC2* locus (bottom right corner), approximate position and the type of the mutation are shown.

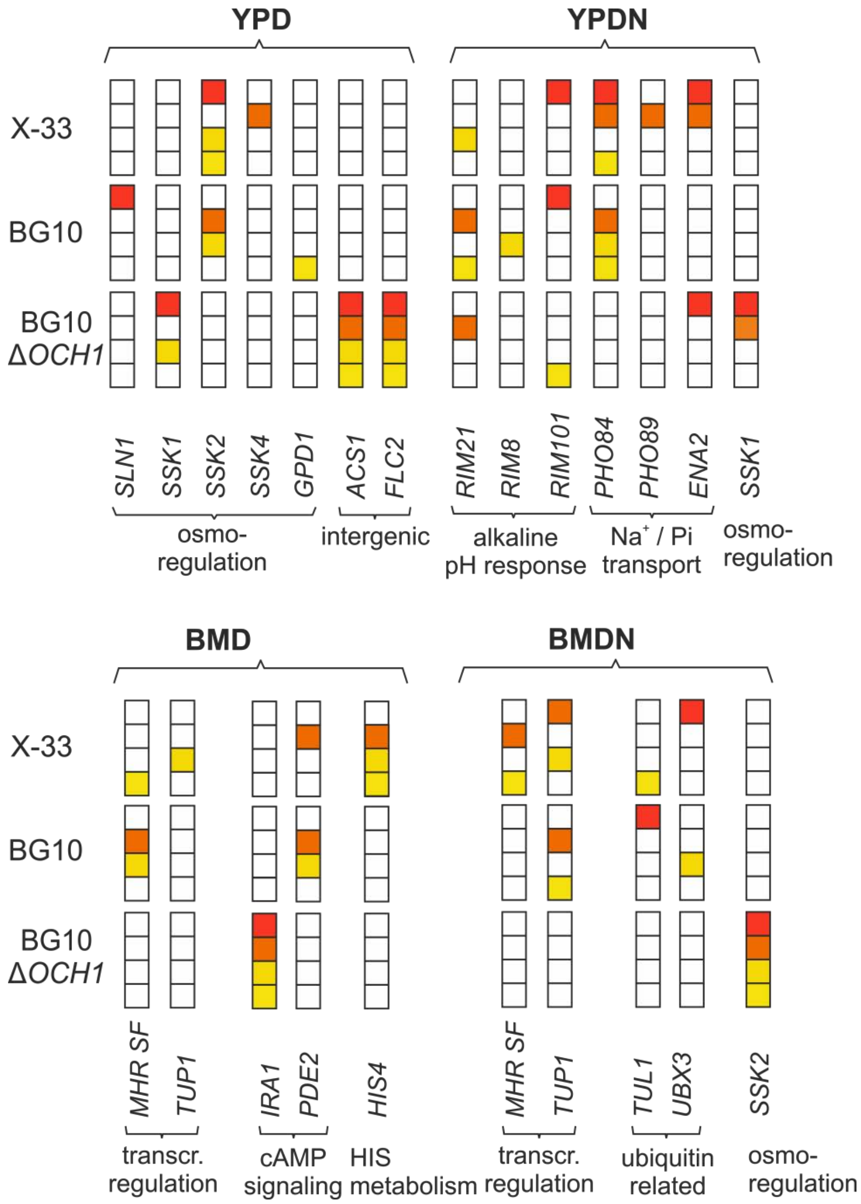

Figure S9. Recurrent mutational targets group by their appearance in strain background and growth condition. For each strain and growth condition recurrent functional modules and the affected genes are shown. Since for each growth condition 4 clones were sequenced (represented by four squares), the occurrence of a particular mutation is indicated by color (yellow to red for the presence of a mutation in each individual clone). A blank square indicates the absence of a mutation. MHR-SF: PAS-ch3_0669 middle homology region superfamily GAL4-like transcriptional regulator.

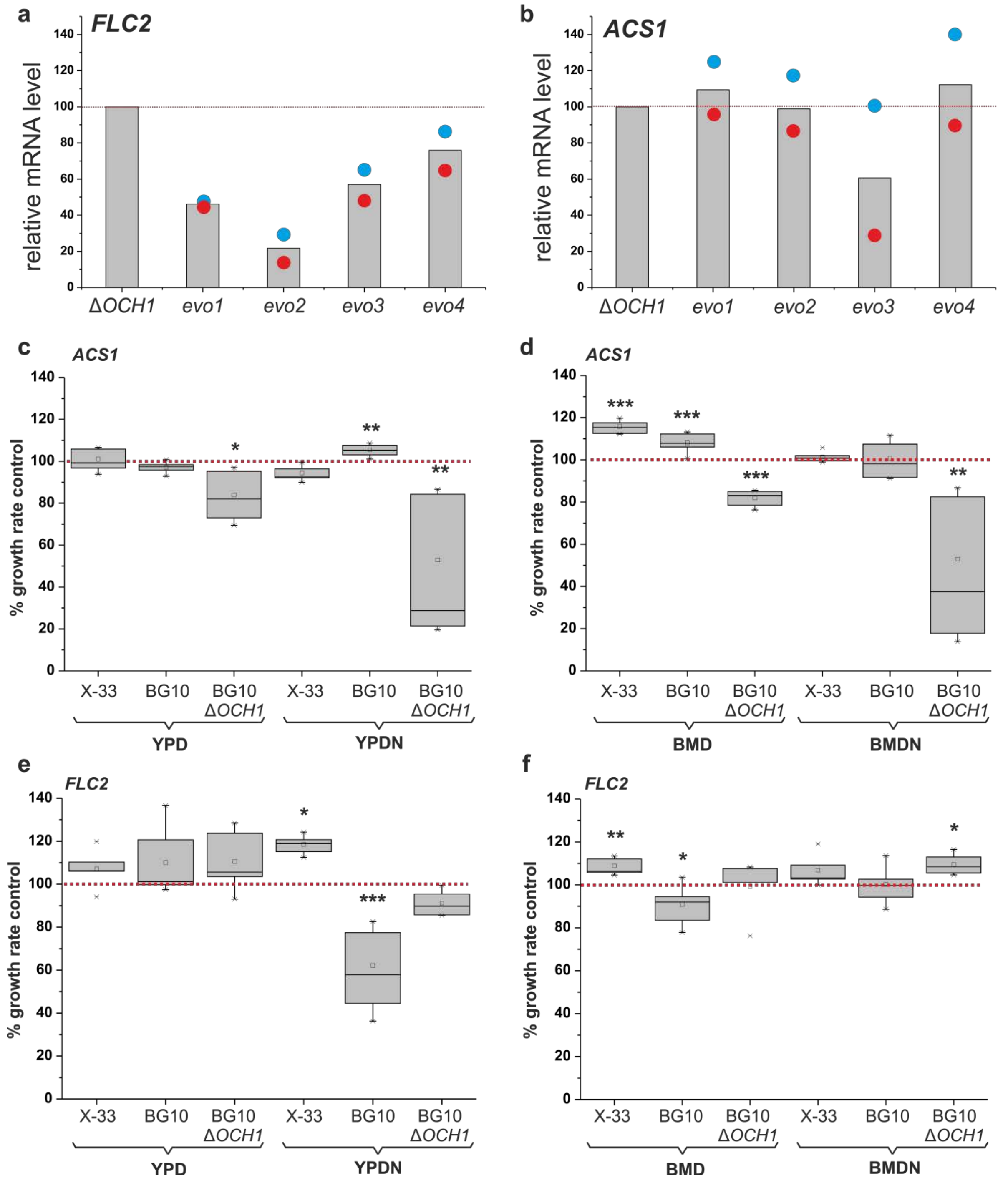

Figure S10. Relative *FLC2* (a) and *ACS1* (b) mRNA expression levels (*ACT1*-normalized) in evolved clones (evo1-4) as compared to the ancestral Δ*OCH1* clone during growth on YPD. The average mRNA level for each strain as well as the individual value for each biological replicate (n = 2) is shown. (c, d) Effect of *ACS1* overexpression in the ancestral strains relative to an empty vector control. (e, f) Effect of *FLC2* overexpression in the ancestral strains relative to an empty vector control. n = 6; paired Student's T-test values * $p \leq 0.05$, ** $p \leq 0.01$ and *** $p \leq 0.001$.

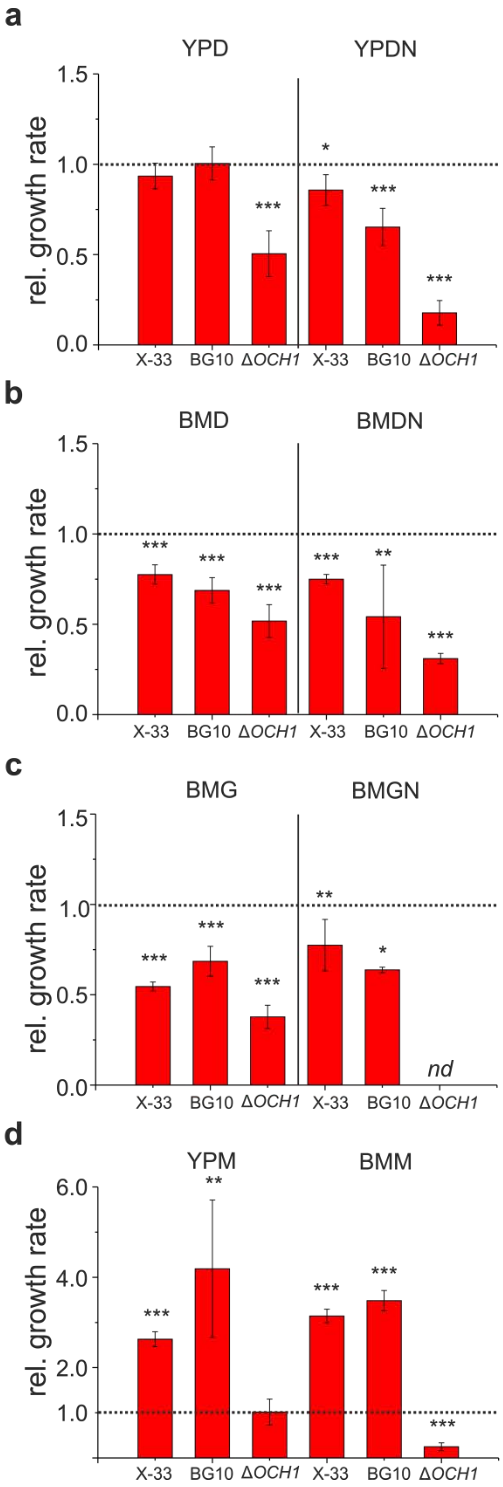

Figure S11. Effect of *TUP1* overexpression in all ancestral *P. pastoris* backgrounds. Growth rates relative to an empty-vector control are shown in various growth media (a) YPD and YPDN (b) BMD and BMDN (c) BMG and BMGN minimal media with glycerol as carbon source and (d) YPM and BMM rich and minimal media with methanol as carbon source. Values represent averages of $n = 6$ +/- standard deviation; paired Student's T-test values * $p \leq 0.05$, ** $p \leq 0.01$ and *** $p \leq 0.001$. *nd* - no growth detected.

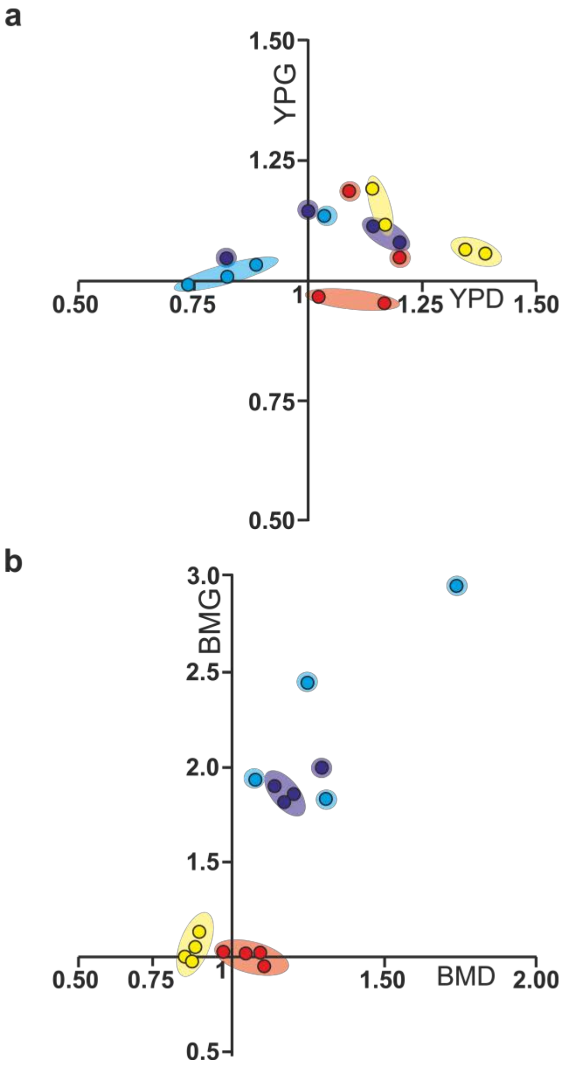

Figure S12. Growth rates of evolved X-33 populations on glucose (x-axis) and glycerol-containing (y-axis) growth media. (a) YPD and YPG growth rates (b) BMD and BMG growth rates. Plots show growth rates of all 4 replicate populations evolved on: YPD (yellow), YPDN (red), BMD (blue) and BMDN (dark blue).

References

1. Stadlmayr G, Mecklenbräuker A, Rothmüller M, Maurer M, Sauer M, Mattanovich D, Gasser B: **Identification and characterisation of novel *Pichia pastoris* promoters for heterologous protein production**. *J Biotechnol* 2010, **150**(4):519-529.
2. Wu G, Yan Q, Jones JA, Tang YJ, Fong SS, Koffas MA: **Metabolic Burden: Cornerstones in Synthetic Biology and Metabolic Engineering Applications**. *Trends Biotechnol* 2016, **34**(8):652-664.
3. Jordà J, Jouhten P, Cámara E, Maaheimo H, Albiol J, Ferrer P: **Metabolic flux profiling of recombinant protein secreting *Pichia pastoris* growing on glucose:methanol mixtures**. *Microb Cell Fact* 2012, **11**:57.
4. Krainer FW, Gmeiner C, Neutsch L, Windwarder M, Pletzenauer R, Herwig C, Altmann F, Glieder A, Spadiut O: **Knockout of an endogenous mannosyltransferase increases the homogeneity of glycoproteins produced in *Pichia pastoris***. *Sci Rep* 2013, **3**:3279.
5. Choi BK, Bobrowicz P, Davidson RC, Hamilton SR, Kung DH, Li H, Miele RG, Nett JH, Wildt S, Gerngross TU: **Use of combinatorial genetic libraries to humanize N-linked glycosylation in the yeast *Pichia pastoris***. *Proc Natl Acad Sci U S A* 2003, **100**(9):5022-5027.
